# Auricular Transcutaneous Vagus Nerve Stimulation Enhances Post‐Stroke Neurological and Cognitive Recovery in Mice by Suppressing Ferroptosis Through α7 Nicotinic Acetylcholine Receptor Activation

**DOI:** 10.1111/cns.70439

**Published:** 2025-05-16

**Authors:** Hongyan Gong, Fang Zheng, Bochao Niu, Bin Wang, Lin Xu, Yunchao Yang, Jiahan Wang, Xiaopeng Tang, Yanlin Bi

**Affiliations:** ^1^ MOE Key Laboratory for Neuroinformation, School of Life Science and Technology University of Electronic Science and Technology of China Chengdu China; ^2^ Department of Anesthesiology, Qingdao Municipal Hospital Qingdao Hospital of Health and Rehabilitation Sciences University Qingdao China; ^3^ Department of Imaging Center, Qingdao Municipal Hospital Qingdao Hospital of Health and Rehabilitation Sciences University Qingdao China; ^4^ Department of Laboratory Animal Science, College of Basic Medical Sciences Qingdao University Qingdao China

**Keywords:** α7 nicotinic acetylcholine receptor, auricular transcutaneous vagus nerve stimulation, ferroptosis, neurogenesis, neuroinflammation

## Abstract

**Aims:**

Ferroptosis plays a critical role in stroke pathophysiology, yet its dynamics during recovery remain unclear. This study aimed to investigate the evolution of ferroptosis throughout post‐stroke recovery and evaluate auricular transcutaneous vagus nerve stimulation (atVNS) as a therapeutic intervention, focusing on the involvement of α7 nicotinic acetylcholine receptor (α7nAChR)‐mediated mechanisms.

**Methods:**

Using a middle cerebral artery occlusion (MCAO) mouse model, we examined ferroptosis‐related protein expression (GPX4, ACSL4, TfR) and iron levels across acute to chronic recovery phases. The therapeutic effects of atVNS were evaluated through the assessment of ferroptosis markers, neurogenesis, angiogenesis, cognitive function, and neuroinflammation. α7nAChR knockout mice were used to investigate the receptor's role in atVNS‐mediated recovery.

**Results:**

We observed sustained alterations in ferroptosis markers and iron levels throughout post‐stroke recovery. atVNS treatment reduced ferroptosis progression by modulating GPX4 and ACSL4 expression, enhanced neurogenesis and angiogenesis, improved cognitive recovery, and reduced neuroinflammation. These beneficial effects were absent in α7nAChR knockout mice, while atVNS increased neuronal α7nAChR expression in wild‐type mice.

**Conclusions:**

This study reveals the persistent involvement of ferroptosis in stroke recovery and demonstrates that atVNS provides comprehensive neuroprotection through α7nAChR‐dependent mechanisms. These findings establish atVNS as a promising noninvasive therapeutic approach for stroke recovery and highlight α7nAChR signaling as a potential therapeutic target.

## Graphical abstract

We reveal that ferroptosis persists from acute to chronic phases after stroke, and auricular vagus nerve stimulation (atVNS) promotes stroke recovery by suppressing ferroptosis through α7 nicotinic acetylcholine receptor activation, providing a promising noninvasive therapeutic strategy for stroke recovery.

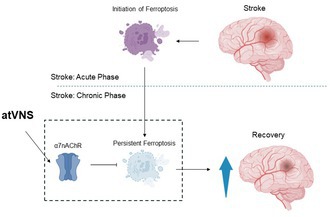

## 1 Introduction

Stroke represents the second leading cause of mortality among adults globally and the primary etiology of permanent disability [1, 2]. Currently, recombinant tissue plasminogen activator (tPA) stands as the sole thrombolytic agent approved by the U.S. Food and Drug Administration (FDA) for clinical stroke management [3]. However, tPA's therapeutic efficacy and safety profile are significantly constrained by its narrow therapeutic window and associated adverse effects [3]. These limitations underscore the imperative for enhanced strategies to improve neurological recovery following cerebrovascular insult, necessitating comprehensive investigation of neurovascular remodeling mechanisms subsequent to cerebral ischemia.

Contemporary research has elucidated the critical role of ferroptosis and aberrant iron metabolism in neuronal death and consequent brain damage during the acute phase of stroke [4, 5, 6, 7]. Within this phase, peri‐infarct tissues manifest iron overload, concomitant with diminished expression of the anti‐ferroptotic molecule glutathione peroxidase 4 (GPX4) and elevated levels of the pro‐ferroptotic protein long‐chain acyl‐CoA synthetase family member 4 (ACSL4) [7, 8, 9]. Ferroptosis extends beyond its association with cellular demise and mitochondrial dysfunction to exhibit intricate interactions with neuroinflammatory processes, notably through modulation of microglial activation and inflammatory signaling cascades (e.g., p38MAPK) [8, 10]. A recent investigation of myocardial infarction, an analogous ischemic condition, demonstrated persistent ferroptosis throughout the recovery phase with significant implications for tissue remodeling and inflammatory responses, particularly via regulation of macrophage polarization [11]. In the context of stroke, conditions characterized by iron overload and elevated transferrin receptor (TfR) expression can persist up to 28 days post‐ictus (i.e., the chronic phase) [12], suggesting potential mechanistic links between ferroptosis, its associated signaling molecules, and the regulation of brain function during stroke recovery. Nevertheless, efficacious interventional strategies targeting ferroptosis during stroke recovery remain to be established.

Vagus nerve stimulation (VNS) has achieved clinical recognition as an adjunctive therapeutic approach for refractory epilepsy [13] and major depression [14], while demonstrating potential for enhancing cognitive function [15, 16]. Preclinical studies indicate that invasive VNS can attenuate neuroinflammation and reduce cerebral infarction volume following stroke through activation of α7 nicotinic acetylcholine receptors (α7nAChR) [16, 17, 18]. However, the invasive nature of conventional VNS techniques limits their application during stroke recovery. Notably, although the vagus nerve primarily comprises sensory afferent fibers from visceral organs and surface areas (approximately 80%) [19], the external ear—specifically the concha region—receives full innervation from the auricular branch of the vagus nerve, thereby constituting an optimal site for stimulation [4]. Consequently, noninvasive auricular transcutaneous vagus nerve stimulation (atVNS), utilizing the external ear as the stimulation target, has garnered significant scientific interest. Nevertheless, the efficacy of atVNS in facilitating neural recovery following stroke remains inadequately characterized.

The present investigation sought to elucidate three fundamental aspects: the temporal dynamics of ferroptosis following cerebral ischemia, the therapeutic potential of atVNS to mitigate ferroptosis and enhance neurological recovery during chronic post‐stroke periods, and the mechanistic role of α7nAChR in mediating the neuroprotective effects of atVNS. Our findings demonstrate persistent ferroptotic activation spanning from acute through chronic recovery phases following stroke. Significantly, atVNS intervention substantially reduced ferroptosis progression while concurrently promoting neurogenesis, angiogenesis, and functional recovery, and attenuating neuroinflammation during chronic stroke recovery. These beneficial outcomes were predominantly mediated by α7nAChR, as evidenced by the markedly diminished therapeutic efficacy of atVNS in α7nAChR knockout mice across multiple recovery parameters.

## 2 Materials and Methods

### 2.1 Animals

Adult male C57BL/6J mice (8–9 weeks old, 22–25 g) were obtained from Qingdao Daren Fucheng Animal Co. Ltd. The animals were maintained under controlled environmental conditions (20°C–22°C, 12‐h light/dark cycle) with ad libitum access to food and water. To elucidate the function of α7nAChR, C57BL/6Smoc‐Chrna7emSmoc (α7nAChR‐Knockout) mice (catalog number NM‐KO‐210112) were sourced from Shanghai Model Organisms Center Inc. All animal experiments adhered to ARRIVE guidelines and were approved by the Qingdao University Laboratory Animal Welfare Ethics Committee (Approval Number: No. 202301C575420240908).

### 2.2 Mouse Middle Cerebral Artery Occlusion Model (MCAO)

The MCAO mouse model was constructed as follows: Mice were anesthetized with 2% isoflurane, and a midline neck incision was made to expose the left common carotid artery (CCA), external carotid artery (ECA), and internal carotid artery (ICA). The proximal CCA and distal ECA were ligated, and an MCAO suture (head diameter: 0.23 mm, body diameter: 0.1 mm, length: 3 cm, silicone‐coated head) was inserted through the ruptured ECA end and advanced along the left ICA to occlude the left middle cerebral artery. After one hour of occlusion, the suture was removed to restore blood flow. Sham‐operated mice underwent identical surgical procedures without suture insertion. Throughout the surgery and two‐hour postoperative recovery period, the mice's core body temperature was maintained at 37°C using heating pads.

### 2.3 Auricular Vagus Nerve Stimulation (atVNS)

Following the MCAO surgery, auricular vagus nerve stimulation (atVNS) was executed on the mice once daily from day 3 (D3) to D7 [4, 20]. This specific treatment window was strategically chosen to balance stroke pathophysiology and recovery dynamics. By initiating atVNS at D3, we avoided potential complications during the acute phase (D0–D3) characterized by rapid pathological processes and allowed for stabilization of the initial injury. The D3–D7 window aligns with the onset of endogenous neurorepair processes in the early subacute phase, potentially amplifying mechanisms such as neuroplasticity and angiogenesis. This timing also harmonizes with clinical rehabilitation timelines, enhancing translational relevance. During the atVNS procedure, the mice were anesthetized for a period of 30 min with isoflurane at concentrations of 2% for induction and 1.5% for maintenance, along with a supply of oxygen at 1 L/min. A heating pad was employed throughout anesthesia to sustain normothermia.

To circumvent cardiac complications, the needle‐like electrode was strategically positioned at the left ear's auricle under atVNS conditions, as illustrated in Figure 2B. This precaution was due to the right branch of the vagus nerve's control over the sinoatrial node, which could potentially induce adverse impact on heart rate [21]. In the context of “Sham” conditions, anesthesia was administered to the mice yet without any electrical stimulation. The stimulation parameters were set as follows: rectangular biphasic pulses, 1 mA intensity, a frequency of 20 pulses per second, with a cycle of 30 s “on” followed by a 5 min “off” period (YC‐3 Bipolar Programmable Stimulator, Chengdu Instruments Factory, Chengdu, China). The total duration was maintained at 30 min, and the pulse width was adjusted to 330 ms.

### 2.4 Experimental Design

Our study is systematically segmented into three distinct sections, each designed with a specific purpose to address our research questions.

**FIGURE 1:**
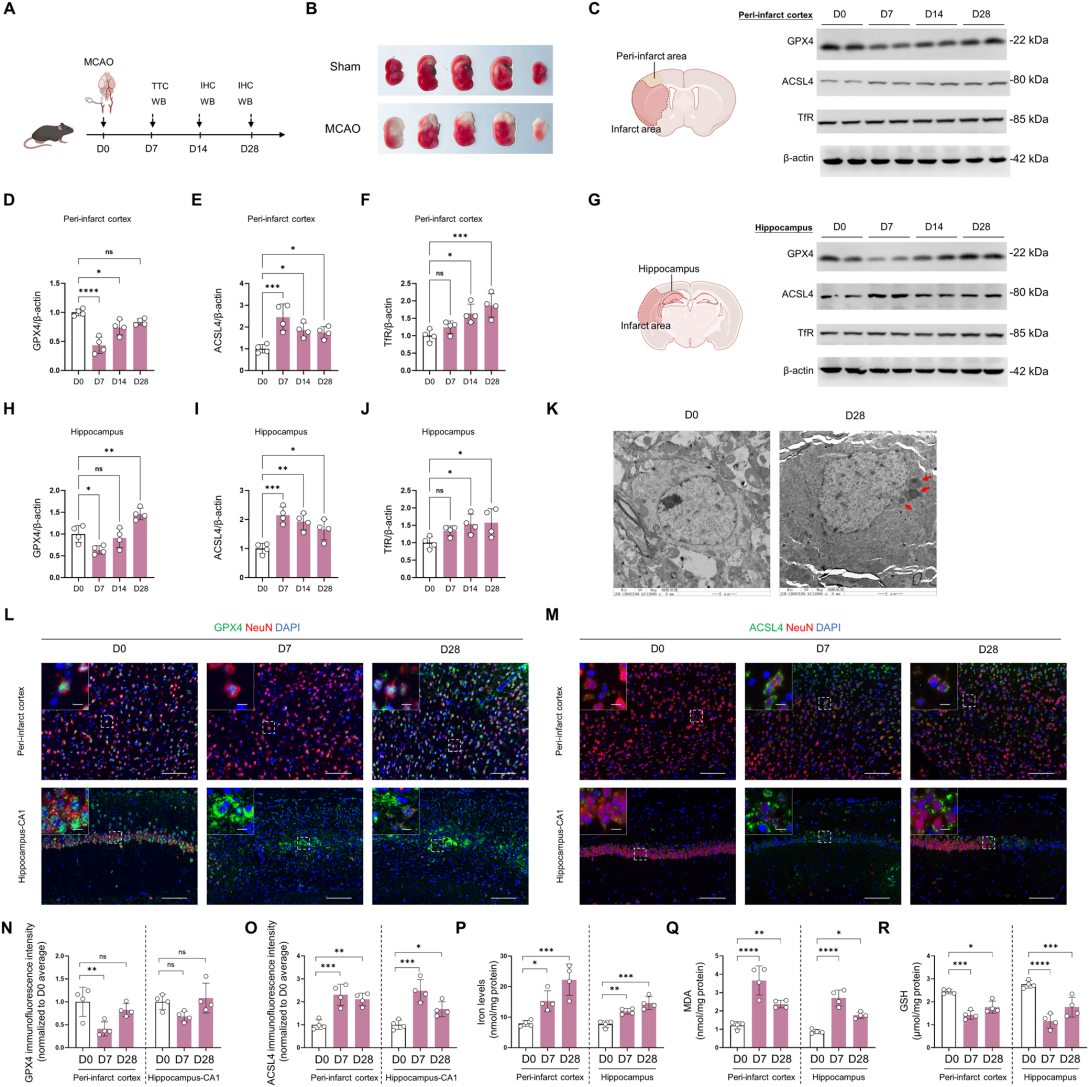
Temporal and spatial dynamics of ferroptosis‐related proteins and iron levels after cerebral ischemia. (A) Experimental timeline for the MCAO (middle cerebral artery occlusion) model and subsequent analyses. (B) Representative TTC (2,3,5‐triphenyltetrazolium chloride) staining of brain sections from sham and MCAO groups at D3 post‐surgery. (C–J) Western blot analysis of GPX4, ACSL4, and TfR expression in the peri‐infarct cortex (C–F) and hippocampus (G–J) at different time points after MCAO. β‐Actin serves as a loading control. Quantification of protein levels normalized to β‐Actin is shown. (K) Transmission electron microscopy images of the peri‐infarct cortex at D0 and D28 post‐MCAO, showing ultrastructural changes indicative of ferroptosis. Scale bars: 2 μm. Red arrows in the D28 panel highlight key features of ferroptosis, including mitochondrial swelling and deformation, increased membrane density, and disruption of organelle structures. The D0 panel shows normal cellular ultrastructure for comparison. (L–O) Immunofluorescence staining and quantification of GPX4 (green) (L,N) and ACSL4 (green) (M,O) in the peri‐infarct cortex and hippocampus‐CA1 region at D0, D7, and D28 post‐MCAO. NeuN (red) marks neurons, DAPI (blue) labels nuclei. Scale bars: 100 μm For main images, 10 μm for insets. Quantification of fluorescence intensity is shown. (P) Quantification of iron levels in the peri‐infarct cortex and hippocampus at D0, D7, and D28 post‐MCAO. (Q, R) Quantification of MDA (malondialdehyde) and GSH (glutathione) levels in the peri‐infarct cortex and hippocampus at D0, D7, and D28 post‐MCAO. Data are presented as mean ± SD. Statistical significance is indicated: *P < 0.05, **p < 0.01, ***p < 0.001, ****p < 0.0001, ns (not significant). Statistical analysis was performed using one‐way ANOVA followed by Tukey's post hoc test (*n =* 4 per group).

The first part of the study, illustrated by Figure 1A, concentrates on the examination of the temporal variations of ferroptosis‐related proteins in the peri‐infarct region and the hippocampus. This design was chosen to establish a baseline understanding of how ferroptosis‐related proteins change over time following a stroke event. By using a single experimental group that underwent MCAO surgery on Day 0 (D0) and conducting measurements on predetermined days, we aimed to create a comprehensive temporal profile of these proteins. This information is crucial for identifying potential therapeutic windows and understanding the progression of ferroptosis‐related processes post‐stroke.

**FIGURE 2:**
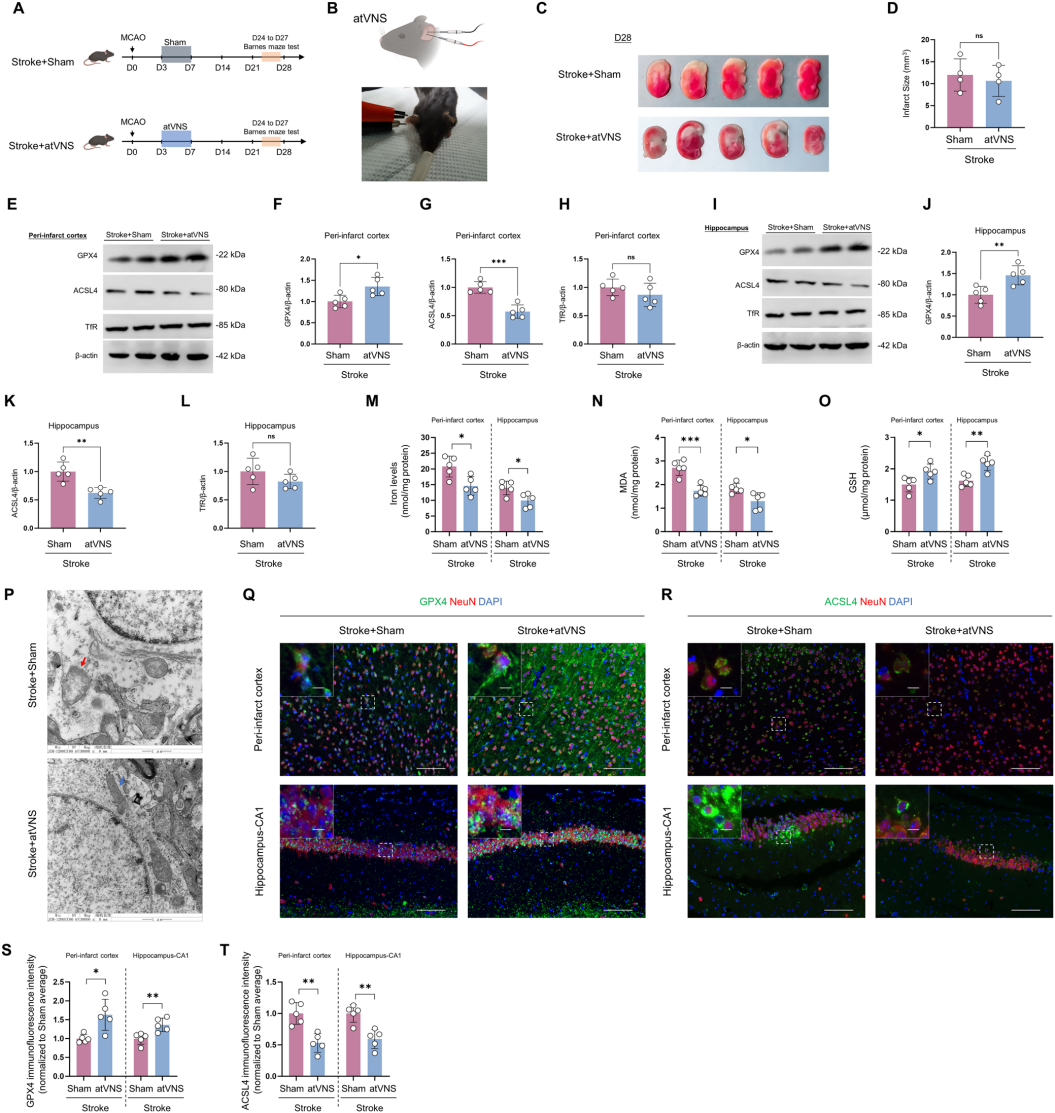
Impacts of auricular vagus nerve stimulation (atVNS) on the modulation of ferroptosis‐related proteins and iron ion concentrations during the chronic recovery phase following stroke. (A) Timeline of the experimental protocol detailing the MCAO model development and subsequent atVNS treatment from D0 to D28, with Barnes maze test conducted between D24 to D27. (B) Schematic diagram accompanied by a photograph demonstrating the atVNS application. (C) Representative TTC staining of brain sections obtained from Stroke+Sham and Stroke+atVNS groups at D28. (D) Analysis showcasing the quantification of the infarct size at D28 (*n =* 5 for each group, ‘ns’ denotes not significant). (E–H) Western blot investigations alongside their respective quantifications for GPX4, ACSL4, and TfR within the peri‐infarct cortex region at D28 (*n =* 5 for each group, *p < 0.05, ***p < 0.001, ‘ns’ indicating not significant). (I–L) Western blot examinations combined with quantification for GPX4, ACSL4, and TfR in the hippocampus at D28 (*n =* 5 for each group, **p < 0.01, ‘ns’ represents not significant). (M) Evaluation of iron ion levels within both the peri‐infarct cortex and hippocampal regions at D28 (*n =* 5 per group, *p < 0.05). (N, O) Quantification of MDA and GSH levels in the peri‐infarct cortex and hippocampus at D28 (*n =* 5 per group, *p < 0.05, **p < 0.01, ***p < 0.001). (P) Transmission electron microscopy of peri‐infarct cortex at D28. Stroke+Sham: Red arrow indicates mitochondrion with ferroptotic features. Stroke+atVNS: Blue arrow indicates mildly swollen mitochondrion with intact cristae. Scale bars: 1 μm. (Q, R) Immunofluorescent staining of GPX4 (green) (Q) and ACSL4 (green) (R) within the peri‐infarct cortex and hippocampal CA1 area at D28. NeuN (red) is utilized to identify neurons, while DAPI (blue) has been employed to label nuclei. The scale bars equal 100 μm for primary images, and 10 μm for the insets. (S, T) Quantification of immunofluorescence intensity for GPX4 (S) and ACSL4 (T) in peri‐infarct cortex and hippocampal CA1 regions (*n =* 5 per group, *p < 0.05, **p < 0.01). Data are exhibited as mean ± SD. Statistical examinations were conducted using either the unpaired t test or Mann–Whitney U test, as deemed appropriate.

The second component, represented in Figure 2A, was crafted to contrast the impacts of sham stimulation versus atVNS post‐MCAO. This design allows us to directly assess the efficacy of atVNS as a potential therapeutic intervention for stroke. By comparing the Stroke+Sham group with the Stroke+atVNS group, we can isolate the effects of atVNS from any potential confounding factors related to the stimulation procedure itself. This comparison is essential for determining whether atVNS offers significant benefits over standard care in stroke recovery.

The third segment, portrayed in Figure 7A, aims to investigate the consequences of atVNS on wild‐type (WT) mice and α7 nicotinic acetylcholine receptor knockout (α7nAChR^−/−^) mice following MCAO. This design was chosen to elucidate the potential mechanism through which atVNS exerts its effects. By including both WT and α7nAChR^−/−^ mice, we can determine whether the α7 nicotinic acetylcholine receptor plays a crucial role in mediating the effects of atVNS. This comparison allows us to test the hypothesis that the beneficial effects of atVNS are dependent on α7nAChR signaling.

The decision to administer atVNS treatment daily to mice from the 3rd to the 7th day post‐stroke was based on several important considerations: (1) Acute phase avoidance: The initial 3 days post‐stroke represent the acute phase, characterized by significant pathological alterations. By delaying atVNS treatment until after this period, we aimed to avoid potential complications and interference with the natural course of acute stroke progression; (2) Stabilization and repair phase: The 3rd to 7th day post‐stroke represents a period when initial damage begins to stabilize and neural repair processes become active. This window provides an opportunity to potentially enhance natural recovery processes through atVNS intervention; (3) Minimizing anesthesia‐related risks: Prolonged and repeated anesthesia could potentially harm the cognitive function of mice. By limiting the treatment period to 5 days, we aimed to balance the need for sufficient intervention with the risk of anesthesia‐related complications.

This experimental design allows us to comprehensively evaluate the temporal dynamics of ferroptosis‐related proteins post‐stroke, assess the efficacy of atVNS as a therapeutic intervention, and investigate the potential mechanistic role of α7nAChR in mediating atVNS effects. By carefully considering the timing of our intervention, we aimed to maximize the potential benefits of atVNS while minimizing confounding factors and risks.

### 2.5 Immunohistochemistry (IHC)

Brain tissues were collected via cardiac perfusion with 4% paraformaldehyde. Coronal sections containing SVZ or hippocampal regions were paraffin‐embedded and sectioned (5 μm thickness). Antigen retrieval was performed by deparaffinization, rehydration, and heating in citrate buffer (pH 6.0). Sections were blocked with 3% BSA and incubated overnight at 4°C with primary antibodies (Table 1). After PBS rinsing, sections were incubated with fluorescently labeled secondary antibodies (FITC or Cy3, 1:200, Servicebio). Nuclei were counterstained with DAPI. Images were acquired using an Olympus BX53 fluorescence microscope (100× magnification) and analyzed with ImageJ software.

**TABLE 1:** Detailed primary antibody information.

Antibody	Host species	Clone type	Manufacturer	Category number	Dilution for WB	Dilution for IHC
ACSL4	Rabbit	Monoclonal	Abclonal	#A20414	1:1000	1:200
CD31	Rabbit	Monoclonal	Abcam	#ab134168	NA	1:400
DCX	Rabbit	Polyclonal	Abcam	#ab18723	NA	1:200
GFAP	Mouse	Monoclonal	Cell Signaling Technology	#3670	NA	1:400
GPX4	Rabbit	Polyclonal	Abclonal	#A1933	1:2000	1:400
Iba‐1	Goat	Polyclonal	Novus Biologicals	#NB100‐1028	NA	1:200
Ki67	Mouse	Monoclonal	Servicebio	#GB121141	NA	1:400
Nestin	Rabbit	Polyclonal	Abclonal	#A11861	NA	1:200
NeuN	Mouse	Monoclonal	Abcam	#ab104224	NA	1:400
p‐ERK	Rabbit	Monoclonal	Cell Signaling Technology	#4370	1:1000	1:200
p‐p38 MAPK	Rabbit	Monoclonal	Cell Signaling Technology	#4511	1:1000	1:200
TfR	Rabbit	Monoclonal	Abclonal	#A25900	1:1000	NA
TNF‐α	Mouse	Monoclonal	Proteintech	# 60291‐1‐Ig	1:1000	NA
α7nAChR	Rabbit	Polyclonal	Servicebio	#GB111335	1:1000	1:200
β‐Actin	Mouse	Monoclonal	Sigma‐Aldrich	#A2228	1:10000	NA

Abbreviations: α7nAChR, Alpha 7 Nicotinic Acetylcholine Receptor; ACSL4, Acyl‐CoA Synthetase Long Chain Family Member 4; DCX, Doublecortin; GFAP, Glial Fibrillary Acidic Protein; GPX4, Glutathione Peroxidase 4; Iba‐1, Ionized calcium‐binding adapter molecule 1; IHC, Immunohistochemistry; Ki67, Marker of proliferation Ki‐67; NA, Not Applicable; NeuN, Neuronal Nuclei; p‐ERK, Phosphorylated Extracellular Signal‐Regulated Kinase; p‐p38 MAPK, Phosphorylated p38 Mitogen‐Activated Protein Kinase; TfR, Transferrin Receptor; TNF‐α, Tumor Necrosis Factor alpha; WB, Western Blot.

### 2.6 Western Blot Analysis

Following euthanasia via intraperitoneal administration of a lethal dose of pentobarbital anesthetic, peri‐infarct cortical and hippocampal tissues were harvested and precisely weighed. Tissue homogenization was performed on ice using RIPA lysis buffer supplemented with protease inhibitor cocktail (#G2006, Servicebio). Protein quantification was conducted utilizing the bicinchoninic acid (BCA) assay kit (#P0012, Beyotime, Shanghai, China). Equivalent protein quantities (20 μg per sample) were resolved by 12% sodium dodecyl sulfate‐polyacrylamide gel electrophoresis (SDS‐PAGE) and subsequently transferred to polyvinylidene difluoride (PVDF) membranes with 0.45 μm pore size (Millipore, Bedford, MA, USA). Membrane blocking was performed for 1 h using either 3% skim milk or bovine serum albumin (BSA) for phosphorylated protein targets, followed by overnight incubation at 4°C with specific primary antibodies (detailed in Table 1). Post‐incubation, membranes underwent three sequential 5‐min washes with Tris‐buffered saline containing 0.1% Tween‐20 (TBST). Subsequently, membranes were incubated for 1 h at ambient temperature with horseradish peroxidase (HRP)‐conjugated goat anti‐rabbit or anti‐mouse immunoglobulin G (IgG) (1:10000 dilution, #AS014 or #AS003, Abclonal, Wuhan, China). Immunoreactive bands were visualized using enhanced chemiluminescence reagent (#BL523B, Biosharp, Anhui, China) and detected via a chemiluminescent imaging system (ImageQuant LAS 4000 mini, GE Healthcare). Densitometric analysis of immunoblots was performed using ImageJ software.

### 2.7 MDA and GSH Detection Methods

For assessment of oxidative stress parameters, lipid peroxidation and glutathione levels were quantified using the malondialdehyde (MDA) Assay Kit (Servicebio, #G4302) and reduced glutathione (GSH) Assay Kit (Servicebio, #G4305), respectively. Briefly, peri‐infarct cortical and hippocampal tissues were homogenized in appropriate lysis buffers and centrifuged at 13,000 x g for 10 min. MDA quantification involved the reaction of supernatant with thiobarbituric acid (TBA) to generate the MDA‐TBA chromophore, with absorbance measured spectrophotometrically at 532 nm using a FlexStation 3 Multi‐Mode Microplate Reader (Molecular Devices, USA). For GSH determination, protein‐free supernatant was reacted with 5,5′‐dithiobis(2‐nitrobenzoic acid) (DTNB) to produce a chromogenic product, with absorbance measured at 412 nm using the aforementioned microplate reader.

### 2.8 Iron Content Analysis

We assessed the total iron content in the hippocampus and cortex utilizing Inductively Coupled Plasma Mass Spectrometry (ICP‐MS). Initially, the tissue samples were weighed and subsequently placed into quartz tubes, to which 2 mL of ultra‐pure nitric acid (65%) was added. The samples underwent digestion in a microwave system at 130°C for 2 h until complete dryness was achieved. For control purposes, 2 mL of concentrated nitric acid was also added to empty tubes under identical conditions. Post‐digestion, the samples were diluted with ultra‐pure water to reach a final volume of 2 mL. Both sample tubes and blank control tubes were then subjected to analysis. The iron content was quantified using a standard calibration curve with a concentration range from 0 to 200 μg/L. Ultimately, the total iron concentration within the tissue was expressed in terms of nmol/mg protein.

### 2.9 Measurement of Cerebral Infarct Volume

For measurement of infarct volume, mice were anesthetized with an overdose intraperitoneal injection of 1% pentobarbital sodium and were then humanely sacrificed at predetermined time points. Following this, coronal brain slices were acquired at 1 mm intervals. These samples were incubated for 30 min in a solution of 2% 2,3,5‐Triphenyltetrazolium Chloride (TTC; Solarbio, Beijing, China; #T8170) at a controlled temperature of 37°C. Subsequently, each brain slice was preserved in a 4% polyformaldehyde solution and photographed for documentation purposes. Trained inspectors, who were blinded to the experimental conditions, utilized Image J software to measure the area of brain infarction. Finally, the percentage of infarct volume was calculated using the formula: (infarct volume on the ipsilateral hemisphere/volume of the contralateral hemisphere) × 100%.

### 2.10 Neurological Assessment

The neurological assessment battery employed in this study comprised the Modified Neurological Severity Score (mNSS) [22, 23], the grid walking test [24, 25], and the pole test [26, 27]. The mNSS is a comprehensive evaluation tool designed to assess motor, sensory, reflex, and balance functions, scored on a scale from 0 to 18, with higher scores indicating greater deficits [22]. To ensure objectivity, all observers were blinded to the experimental group assignments.

Following established protocols [24], the grid walking test was conducted using an iron wire mesh apparatus measuring 300 × 200 × 400 mm, with each square side being 10 mm. A mirror positioned at a 45° angle beneath the mesh facilitated the simultaneous recording of total steps and gait errors or ‘foot‐faults’ during a 5 min observation period. Foot‐faults were defined as stepping actions that failed to provide support, causing the mouse's foot to fall through the grid hole. The relative frequency of foot‐faults was calculated through blind offline analysis using the formula: number of foot‐faults/(number of foot‐faults + number of non‐foot‐fault steps) × 100%, yielding the percentage of foot‐faults.

To evaluate forelimb strength and balance in post‐stroke mice, we administered the pole test on day 28 post‐stroke [26]. Each mouse was placed atop a vertical, rough‐surfaced steel pole, 60 cm in height, with the head facing upwards. We recorded the time taken for the mouse to reorient its body until facing downward (‘time to turn’) and the time taken to reach the bottom with all four paws contacting the ground (‘time to reach the bottom’).

### 2.11 Barnes Maze

The Barnes Maze experiment is predominantly utilized to evaluate spatial learning and memory in mice, with minimal influence from the animals' motor abilities. This makes it an optimal method for assessing cognitive function in post‐stroke mice. The experiment uses a 90‐cm diameter acrylic circular maze with twelve 7 cm diameter holes. The entire procedure, divided into two main segments—the training phase and the probe phase—is recorded and analyzed using ANY‐maze (Stoelting) software.

During the training phase, which lasts four consecutive days, each mouse undergoes three trials per day with 15 min intervals between trials. Initially, the maze is sterilized with a 70% ethanol solution before each trial begins. The mouse is then placed in a black plastic container at the center of the maze. Upon activation of the lighting, the container is removed, allowing the mouse to explore. Only the escape box beneath the target hole remains open while all other holes are locked. The mouse navigates the maze using four visual cues distributed evenly on the perimeter walls. Each trial ends when the mouse successfully enters the escape box or after a 2 min exploration period. If the mouse finds the escape box within this time, the lights are turned off as a reward, and the mouse stays in the box for 1 min. If the mouse fails to find the escape box, it is guided there by the researcher and remains for 1 min. The primary measure during this phase is the latency to reach the escape box.

The probe phase is conducted 24 h after the final training trial. In this phase, the target hole is closed to test the retention of the learned location. The mouse is placed in the central container and allowed to navigate the maze for 90 s. Behavioral indicators during this phase include the time spent in the quadrant previously containing the escape box and the frequency of exploratory errors.

### 2.12 Transmission Electron Microscope

Upon euthanizing the mice, their brains are swiftly removed and sectioned into 1 mm thick slices. These slices are then placed in a solution of 4% glutaraldehyde. Following a thorough cleansing with PBS, the slices undergo fixation via hyperosmotic osmium tetroxide. Subsequently, they are embedded in EPON 812 resin (Sigma‐Aldrich) and further cut into sections measuring 0.06 mm in thickness. The resulting specimens are then stained with uranyl acetate and lead citrate to enhance visibility. Lastly, the ultrastructure of cells surrounding the cerebral ischemia after stroke is examined using a transmission electron microscope.

### 2.13 Statistical Analysis

All data are represented as the mean ± standard deviation (SD). Prior to analysis, all data were subjected to the Shapiro–Wilk test to assess normality. For normally distributed data (*p >* 0.05 on Shapiro–Wilk test), parametric tests were used. For comparisons involving multiple groups with normally distributed data, a one‐way ANOVA supplemented by Tukey's post hoc test was implemented. In instances where two‐group comparisons were evaluated with normally distributed data, unpaired t tests were utilized. For non‐normally distributed data, nonparametric equivalents were employed, such as the Kruskal‐Wallis test for multiple group comparisons and the Mann–Whitney U test for two‐group comparisons. For time‐course analyses, a two‐way ANOVA with Bonferroni's post hoc test was employed for normally distributed data. All statistical evaluations were conducted using GraphPad Prism 6 software (GraphPad Software Inc., USA). A *p*‐value of less than 0.05 was deemed to signify statistical significance.

## 3 Results

### 3.1 Ferroptosis Activation Extends From Acute Phase to Chronic Recovery Period Post‐Stroke

To systematically observe the sequential changes of ferroptosis after cerebral ischemia from the acute phase (D0 and D3) to the chronic phase (D14 and D28), we examined alterations in the expression of ferroptosis‐associated proteins [Glutathione Peroxidase 4 (GPX4), Long‐chain acyl‐CoA synthetase 4 (ACSL4), and Transferrin receptor (TfR)], within the peri‐infarct cortex and hippocampus regions using a cerebral ischemia (MCAO) model (Figure 1A). Our findings showed significant infarction foci in the brain tissue of ischemic mice on the third day post‐cerebral ischemia, as indicated by TTC staining results, thus verifying the successful establishment of the MCAO‐induced cerebral ischemia model (Figure 1B).

Subsequently, we employed Western blot analysis and discerned remarkable shifts in GPX4, ACSL4, and TfR expressions within peri‐infarct cortical and hippocampal areas post‐cerebral ischemia. In the peri‐infarct cortex, the GPX4‐mediated antioxidant defense mechanism experienced impairment during the acute phase of cerebral ischemia, persisting into the chronic phase. Its expression notably diminished at D7 (Figure 1C,D, *p <* 0.0001) and remained at sub‐baseline levels at D14 and D28 (Figure 1C,D, *p <* 0.05, *n =* 4). Contrastingly, within the hippocampal region, GPX4 expression followed a “decrease‐then‐increase” trend post‐cerebral ischemia, being lower at D7 (Figure 1G,H, *p <* 0.05, *n =* 4), but surpassing baseline level by D28 (Figure 1G,H, *p <* 0.01, *n =* 4).

Relative to the baseline, ACSL4 demonstrated an upward trend in both regions post‐ischemia. In the peri‐infarct cortex, ACSL4 expression elevated starting from D7 (Figure 1C,E, *p <* 0.001, *n =* 4), peaking at D14 (*p <* 0.05 vs. D0, *n =* 4). Although it slightly decreased at D28, it maintained above the baseline level (*p <* 0.05, *n =* 4). In the hippocampus, ACSL4 significantly augmented from D7 (Figure 1G,I, *p <* 0.001, *n =* 4) and stayed high at D14 and 28 (both *p <* 0.01 vs. D0, *n =* 4).

Additionally, TfR in both regions also showcased an upward trajectory post‐ischemia. In the peri‐infarct cortex, compared to D0, TfR expression significantly escalated at D14 and D28 (Figure 1F, *p <* 0.05 and *p <* 0.001, *n =* 4). Similarly, in the hippocampus, TfR significantly increased at D14 and D28 (Figure 1G,J, both *p <* 0.05). These observations suggest that the GPX4‐mediated antioxidant mechanism, ACSL4‐driven lipid metabolism, and TfR‐dependent iron ion metabolism underwent considerable alterations following cerebral ischemia in the peri‐infarct cortex and hippocampus. This insinuates that processes associated with ferroptosis are activated not only in the acute phase post‐stroke but may extend into the chronic phase (stroke recovery period).

We observed significant ultrastructural changes in the peri‐infarct cortex at D28 post‐ischemia using transmission electron microscopy (Figure 1K). Key alterations included mitochondrial abnormalities (swelling, shape changes, internal structure disruption, and outer membrane damage), increased cytoplasmic electron density, extensive vacuolization near cell membranes, heightened chromatin density, and reduced organelle numbers. These changes, particularly the mitochondrial pathology, elevated cytoplasmic electron density, and cell membrane abnormalities, strongly indicate persistent ferroptosis following cerebral ischemia.

To corroborate the Western blot findings and delve deeper into the spatiotemporal distribution characteristics of ferroptosis‐related proteins across different brain regions, we employed immunofluorescence technology to visually analyze GPX4 and ACSL4 expressions in the peri‐infarct cortex and hippocampal CA1 region. Immunofluorescence detection results revealed differential expression patterns of GPX4 and ACSL4 in the peri‐infarct cortex and hippocampal CA1 region post‐ischemia. In the peri‐infarct cortex, GPX4 significantly declined during the acute phase post‐cerebral ischemia (Figure 1L,N, comparing with D0, *p <* 0.01 at D7, *n =* 4). It recovered by D28, albeit without any significant difference. Conversely, in the hippocampal CA1 region, GPX4 expression remained relatively stable, with no statistically significant difference (Figure 1L,N, *n =* 4). The expression pattern of ACSL4 in both areas followed an upward trend; however, the dynamics varied significantly. In the peri‐infarct cortex, ACSL4 substantially increased at D7 (Figure 1M,O, *p <* 0.001, *n =* 4), remaining elevated at D28 (*p <* 0.01 vs. D0, *n =* 4). In the hippocampal CA1 region, ACSL4 exhibited a gradual incline, beginning from D7 (*p <* 0.001 vs. D0, *n =* 4), peaking at D28 (*p <* 0.05 vs. D0, *n =* 4).

Moreover, as shown in Figure 1P, it is noteworthy that alterations in iron ion levels closely correlate with ferroptosis. In the peri‐infarct cortex, iron ion levels were significantly higher at D7 and D28 than D0 (*p <* 0.05 and *p <* 0.001, *n =* 4). Analogously, in the hippocampal region, iron ion levels significantly escalated at D7 and D28 relative to D0 (both *p <* 0.001, *n =* 4). Furthermore, we assessed levels of malondialdehyde (MDA), an indicator of lipid peroxidation, and glutathione (GSH), a crucial antioxidant depleted during ferroptosis. In the peri‐infarct cortex, MDA levels were significantly elevated at both D7 and D28 compared to D0 (Figure 1Q, *p <* 0.0001 for both, *n =* 4). Concurrently, GSH levels were markedly reduced at D7 (*p <* 0.001, *n =* 4) and D28 (*p <* 0.0001, *n =* 4) relative to baseline (Figure 1R). Similarly, in the hippocampus, MDA levels increased significantly at D7 (*p <* 0.0001, *n =* 4) and remained elevated at D28 (*p <* 0.05, *n =* 4) compared to D0 (Figure 1Q). Hippocampal GSH levels were also significantly diminished at D7 (*p <* 0.001, *n =* 4) and D28 (*p <* 0.0001, *n =* 4) compared to baseline (Figure 1R).

### 3.2 atVNS Mitigates Progression of Ferroptosis During Chronic Recovery Phase Following Stroke

Western blot analysis revealed that atVNS significantly modulates the expression of ferroptosis‐related proteins in the peri‐infarct cortex and hippocampal region after an ischemic event. Specifically, within the peri‐infarct cortex region, atVNS markedly upregulated GPX4 expression (Figure 2F, *n =* 5, *p <* 0.05) and distinctly downregulated ACSL4 expression (Figure 2G, *n =* 5, *p <* 0.001). In contrast, the expression level of TfR did not exhibit any significant difference between the atVNS and Sham groups (Figure 2H, *n =* 5, *p >* 0.05). Similar trends, albeit more pronounced, were observed in the hippocampus: GPX4 expression was significantly elevated (Figure 2J, *n =* 5, *p <* 0.01), and ACSL4 expression was significantly reduced (Figure 2K, *n =* 5, *p <* 0.01) following atVNS treatment. Again, there was no statistical difference in TfR expression between the two groups (Figure 2L, *n =* 5, *p >* 0.05). Moreover, measurements of iron ion levels (Figure 2M) demonstrated that atVNS considerably reduces iron ion concentrations in the peri‐infarct cortex and hippocampal regions (*n =* 5, both *p <* 0.05).

Analysis of oxidative stress markers further supported the anti‐ferroptotic effects of atVNS. Measurements of malondialdehyde (MDA) levels (Figure 2N) revealed that atVNS treatment significantly decreased lipid peroxidation in both the peri‐infarct cortex (*p <* 0.001) and hippocampus (*p <* 0.05) compared to the Sham group. Conversely, glutathione (GSH) levels (Figure 2O) were significantly elevated following atVNS treatment in both the peri‐infarct cortex (*p <* 0.05) and hippocampus (*p <* 0.01), indicating enhanced antioxidant capacity. These findings suggest that atVNS exerts neuroprotective effects by reducing oxidative stress associated with ferroptosis.

Immunofluorescent staining analysis further corroborated the effect of atVNS on the expression of ferroptosis‐associated proteins. In the peri‐infarct cortex region and hippocampal CA1 region, quantitative analysis (Figure 2S) revealed that the fluorescent intensity of GPX4 in the atVNS treatment group was significantly greater than in the Sham group (peri‐infarct cortex: *p <* 0.05; hippocampus CA1: *p <* 0.01). Contrastingly, ACSL4 expression significantly attenuated post‐atVNS treatment (Figure 2T), with markedly lower fluorescent intensity in both the peri‐infarct cortex and hippocampal CA1 regions compared to the Sham group (both *p <* 0.01). Importantly, alterations in GPX4 and ACSL4 expression were predominantly present in neurons (NeuN‐positive cells), suggesting that the regulatory influence of atVNS on ferroptosis‐related proteins primarily occurs within neurons. Furthermore, electron microscope images of the peri‐infarct cortex region (Figure 2P) reveal more intact morphology and clearer cristae structure in neuronal mitochondria from the atVNS group compared to the Sham group. This observation suggests that atVNS may deter the onset of ferroptosis by maintaining mitochondrial function.

### 3.3 atVNS Enhances Neurological and Cognitive Recovery Post‐Stroke

**FIGURE 3:**
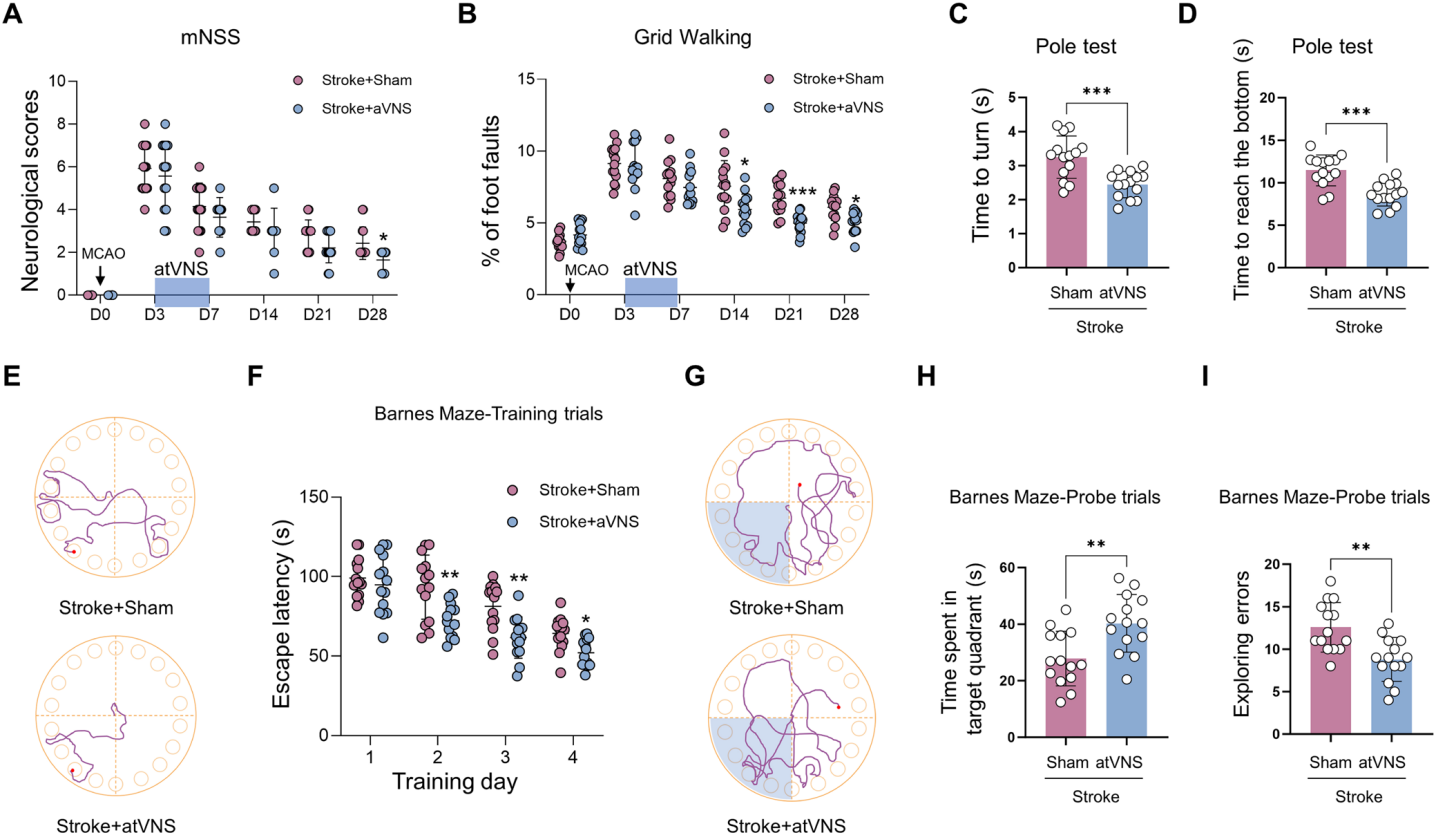
Effects of auricular vagus nerve stimulation (atVNS) on neurological and cognitive functions after stroke. (A) Modified Neurological Severity Scores (mNSS) from D0 to D28 post‐MCAO for Stroke+Sham and Stroke+atVNS groups (*n =* 14 per group, *p < 0.05). (B) Grid Walking test results showing the percentage of foot faults from D0 to D28 (*n =* 14 per group, *p < 0.05, ***p < 0.001). (C, D) Pole test results on D28, showing the time to turn (C) and the time to reach the bottom (D) (*n =* 14 per group, ***p < 0.001). (E) Representative traces of mouse movement in the Barnes Maze during training trials for Stroke+Sham and Stroke+atVNS groups. (F) Escape latency in Barnes Maze training trials over 4 days (*n =* 14 per group, *p < 0.05, **p < 0.01). (G) Representative traces of mouse movement in the Barnes Maze during probe trials. (H–I) Barnes Maze probe trial results showing the time spent in the target quadrant (H) and the number of exploring errors (I) (*n =* 14 per group, **p < 0.01). Data are presented as mean ± SD. Statistical analysis was performed using two‐way ANOVA with Bonferroni's *post hoc* test for A, B, and F, and unpaired t test for C, D, H, and I.

To assess the efficacy of atVNS on post‐stroke neurological and cognitive functions, we conducted a series of behavioral tests on two experimental groups: Stroke+Sham and Stroke+atVNS (*n =* 14). The modified Neurological Severity Scores (mNSS, Figure 3A) revealed that both groups exhibited baseline scores of zero prior to the Middle Cerebral Artery Occlusion (MCAO) procedure (D0), with scores peaking at approximately 6 points on D3. Notably, the atVNS group demonstrated a more rapid recovery trajectory, achieving significantly lower scores than the Sham group by D28 (*p <* 0.05). This outcome suggests that atVNS may facilitate enhanced post‐stroke neurological function recovery. The Grid Walking tests conducted on D3 (Figure 3B) initially showed substantial walking impairments in both groups, evidenced by foot faults of approximately 9%. However, from D14 onwards, the atVNS group exhibited marked improvement, surpassing the performance of the Sham group (*p <* 0.05). This enhanced performance persisted through D28 (*p <* 0.05), indicating a significant improvement in motor coordination facilitated by atVNS. Further corroborating the beneficial effects of atVNS, the Pole test results (Figure 3C,D) demonstrated superior performance in the atVNS group on D28. This was characterized by significant reductions in both turning time (Figure 3C, *p <* 0.001) and time taken to reach the base (Figure 3D, *p <* 0.001), suggesting notable improvements in balance and fine motor control attributable to atVNS.

Cognitive function evaluation using the Barnes Maze tests revealed intriguing results. During the training phase (Figure 3E,F), the atVNS group exhibited significantly shorter escape latency periods on days 2, 3, and 4 compared to the Sham group (*p <* 0.01, *p <* 0.01, *p <* 0.05, respectively). This observation provides evidence for the role of atVNS in enhancing spatial learning capabilities. Moreover, the exploration experiment results (Figure 3G–I) demonstrated that the atVNS group spent significantly more time in the target quadrant relative to the Sham group (Figure 3H, *p <* 0.01) and committed fewer exploration errors (Figure 3I, *p <* 0.01). These findings collectively indicate a profound improvement in spatial memory ability, further supporting the efficacy of atVNS in cognitive recovery post‐stroke.

### 3.4 atVNS Promotes Neurogenesis and Angiogenesis Following Stroke

The significant roles of neurogenesis and angiogenesis in the neurological recovery following brain ischemia, typically experienced post‐stroke, have been well documented. In our current study, we examine the impact of atVNS on these two processes, utilizing immunofluorescent staining analyses with Stroke+Sham and Stroke+atVNS cohorts. Our primary focus is on the subventricular zone (SVZ) and the dentate gyrus (DG), known locations for neurogenesis, along with the peri‐infarct cortex, a site of interest for angiogenesis.

**FIGURE 4:**
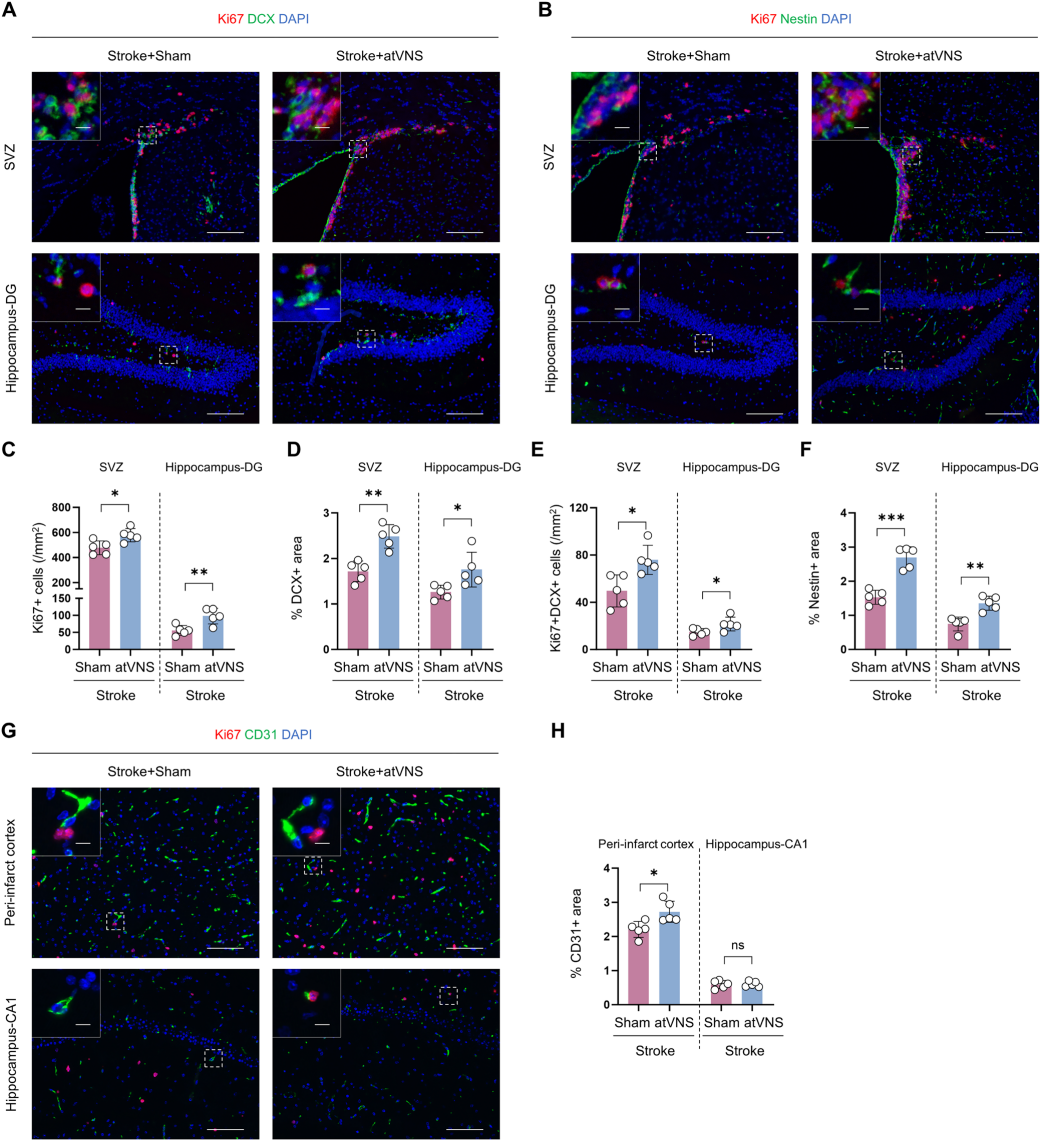
Effects of auricular vagus nerve stimulation (atVNS) on neurogenesis and angiogenesis during the recovery period after stroke. (A) Representative immunofluorescence images of Ki67 (red) and DCX (green) staining in the subventricular zone (SVZ) and hippocampus dentate gyrus (DG) for Stroke+Sham and Stroke+atVNS groups. DAPI (blue) labels nuclei. Scale bars: 100 μm for main images, 10 μm for insets. (B) Representative immunofluorescence images of Ki67 (red) and Nestin (green) staining in the SVZ and DG. Scale bars: 100 μm for main images, 10 μm for insets. (C) Quantification of Ki67+ cells in the SVZ and DG (*n =* 5 per group, *p < 0.05, **p < 0.01). (D) Quantification of DCX+ area percentage in the SVZ and DG (*n =* 5 per group, *p < 0.05, **p < 0.01). (E) Quantification of Ki67 + DCX+ cells in the SVZ and DG (*n =* 5 per group, *p < 0.05). (F) Quantification of Nestin+ area percentage in the SVZ and DG (*n =* 5 per group, **p < 0.01, ***p < 0.001). (G) Representative immunofluorescence images of Ki67 (red) and CD31 (green) staining in the peri‐infarct cortex and hippocampus CA1 region. Scale bars: 100 μm for main images, 10 μm for insets. (H) Quantification of CD31+ area percentage in the peri‐infarct cortex and hippocampus CA1 region (*n =* 5 per group, *p < 0.05, ns: Not significant). Data are presented as mean ± SD. Statistical analysis was performed using unpaired t test.

With respect to neurogenesis, we observed a significant increase in Ki67+ cells in both the SVZ and DG in response to atVNS (Figure 4A,C,E; *p <* 0.05, *n =* 5). Specifically, approximately 500 such cells/mm [2] were noted in the SVZ of the atVNS group, compared to about 400 cells/mm^2^ in the Sham group (*p <* 0.05, *n =* 5). In the DG, the atVNS cohort presented approximately 100 Ki67+ cells/mm^2^, contrasted with around 50 cells/mm^2^ in the Sham group (*p <* 0.01, *n =* 5). These results suggest that atVNS potently stimulates the proliferation of neural precursor cells.

Furthermore, an elevated percentage of DCX+ regions was detected following atVNS treatment in both the SVZ and DG (Figure 4A,D). In the SVZ, the atVNS group exhibited around 2.5% DCX+ regions, compared to about 1.8% in the Sham group (*p <* 0.01, *n =* 5). A similar trend was observed in the DG, with approximately 1.8% for the atVNS group and 1.2% for the Sham group (*p <* 0.05, *n =* 5). The marked increase in Ki67+/DCX+ dual‐positive cells further corroborates the pro‐proliferative effect of atVNS on neural progenitor cells.

Complementing these findings, evaluation of Nestin expression, a recognized marker of neural stem cells (Figure 4B,F), indicated an augmented percentage of Nestin+ zones in the atVNS group relative to the Sham group in both the SVZ and DG. In the SVZ, this percentage rose to about 2.8% for the atVNS cohort compared to approximately 1.8% for the Sham group (*p <* 0.001, *n =* 5). In the DG, comparative values for the atVNS and Sham groups stood at roughly 1.4% and 0.8%, respectively (*p <* 0.01, *n =* 5). These outcomes suggest that atVNS not only enhances the proliferation of neural progenitor cells but also increases the quantity of neural stem cells, potentially establishing a foundation for sustained cell sourcing required for long‐term neural repair.

With regards to angiogenesis, we examined the expression of CD31, a marker for endothelial cells (Figure 4G,H). A noticeable increase in the percentage of CD31+ regions was observed within the peri‐infarct cortex in the atVNS group when compared with the Sham group, with reported figures of approximately 2.7% and 2.3%, respectively (*p <* 0.05, *n =* 5). This suggests that atVNS contributes to the proliferation of endothelial cells and furthers angiogenesis. Yet, it is worth mentioning that no substantial variation was detected between the groups in terms of the percentage of CD31+ areas within the hippocampal CA1 region (*p >* 0.05, *n =* 5). Additionally, we observed that in our surveyed regions, there were virtually no cells presenting both CD31+ and Ki67+. These region‐specific angiogenic responses potentially underscore the differential effects of atVNS across various brain regions or could be linked to the spatial patterns of ischemic damage.

### 3.5 atVNS Treatment Attenuates Glial Activation and Inflammation‐Related Signaling Pathways During the Post‐Stroke Recovery Period

To elucidate the impact of atVNS on post‐ischemic cerebral inflammation, we conducted both immunofluorescence staining and Western blot analysis on Stroke+Sham and Stroke+atVNS groups. Our investigation primarily centered on alterations in microglial activation, astrocyte proliferation, as well as the modifications in inflammation‐related signaling pathways (p38MAPK and ERK) within the peri‐infarct cortex and hippocampal CA1 regions.

**FIGURE 5:**
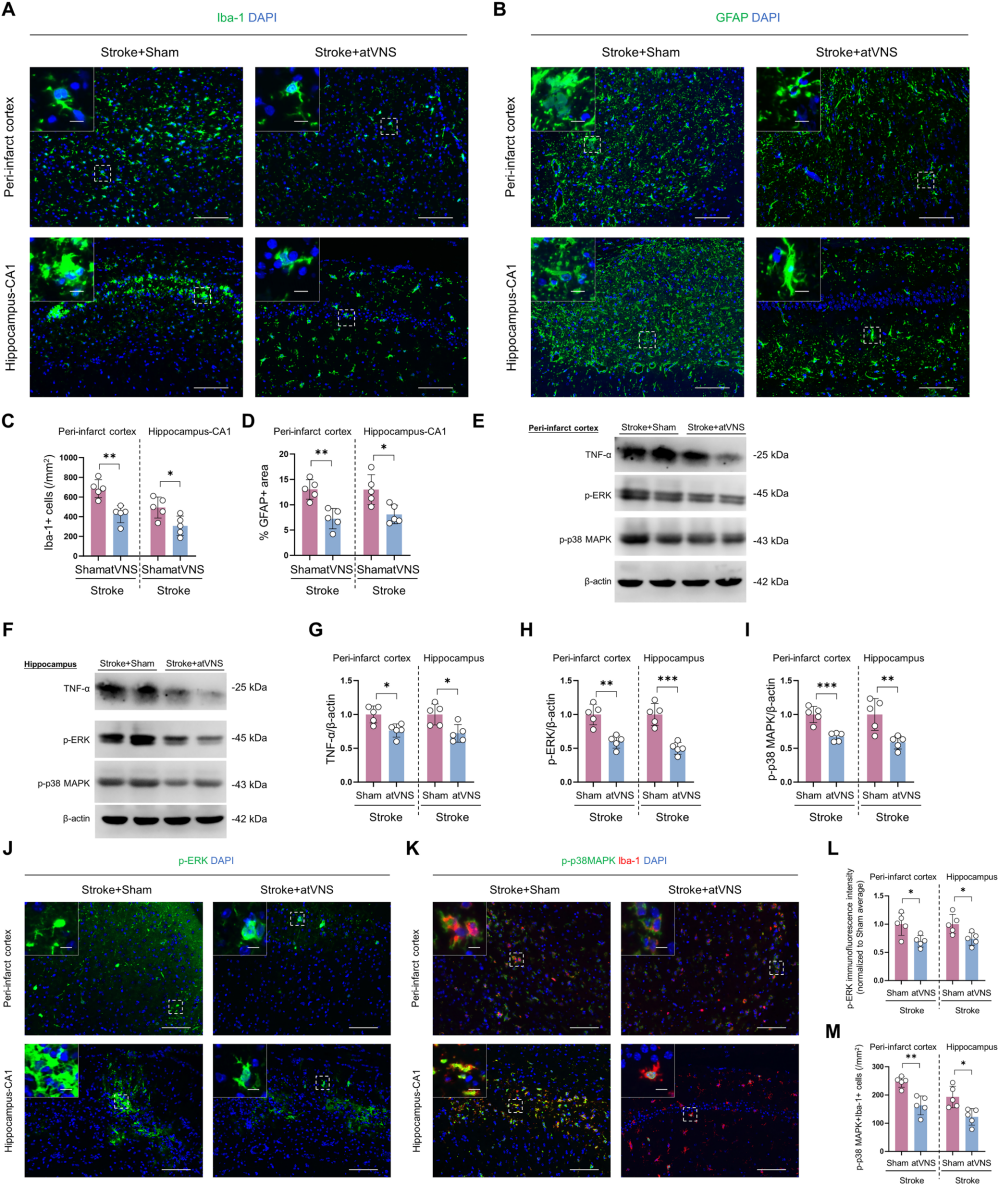
Impact of auricular vagus nerve stimulation (atVNS) on post‐stroke neuroinflammation. (A) Illustrative immunofluorescence images depicting Iba‐1 (green) staining in the peri‐infarct cortex and hippocampal CA1 regions, with DAPI (blue) marking nuclei. Scale bars represent 100 μm for primary images and 10 μm for insets. (B) Representative images showcasing GFAP (green) staining within the same regions. The scale bars are identical to (A). (C) Quantitative representation of Iba‐1 positive cells within the peri‐infarct cortex and hippocampal CA1 areas (*n =* 5 per group, *p < 0.05, **p < 0.01). (D) Analysis of GFAP‐positive area percentages in the aforementioned regions (*n =* 5 per group, *p < 0.05, **p < 0.01). (E, F) Characteristic Western blot images of TNF‐α, p‐ERK, and p‐p38 MAPK in the peri‐infarct cortex (E) and hippocampus (F). (G‐I) Data quantification from Western blot analyses for TNF‐α (G), p‐ERK (H), and p‐p38 MAPK (I), each normalized to β‐Actin (*n =* 5 per group, *p < 0.05, **p < 0.01, ***p < 0.001). (J) Exemplary immunofluorescence images displaying p‐ERK (green) in the peri‐infarct cortex and hippocampal CA1 areas. The scale bars are consistent with (A). (K) Illustrative images of co‐staining for p‐p38 MAPK (green) and Iba‐1 (red) within the same regions. Again, the scale bars remain unchanged. (L) Fluorescence intensity quantification for p‐ERK within the peri‐infarct cortex and hippocampal CA1 sectors (*n =* 5 per group, *p < 0.05). (M) Quantification of p‐p38 MAPK and Iba‐1 co‐labeled cells within these areas (*n =* 5 per group, *p < 0.05, **p < 0.01). All data are expressed as mean ± SD. An unpaired t test was utilized for statistical analyses.

Immunofluorescent staining unveiled a significant inhibition of glial cell response following cerebral ischemia due to atVNS. Within the peri‐infarct cortex region, there was a notable reduction in the count of Iba‐1 positive cells in the atVNS group (approximately 450 per mm^2^) when compared to the Sham group (approximately 700 per mm^2^) (Figure 5A,C, *p <* 0.01, *n =* 5). In parallel, in the hippocampal CA1 region, a substantial reduction in the Iba‐1 positive cells was observed in the atVNS group (roughly 300 per mm^2^), contrasted with the Sham group (around 500 per mm^2^) (*p <* 0.05, *n =* 5). These data suggest that atVNS exerts a potent inhibitory effect on microglial activation.

Simultaneously, the GFAP staining results revealed significant reductions in the percentage of GFAP‐positive areas in both the peri‐infarct cortex and hippocampal CA1 regions for the atVNS group as compared to the Sham group (Figure 5B,D). In the peri‐infarct cortex region specifically, the percentage of GFAP‐positive area was only about 7% in the atVNS group as opposed to roughly 12% in the Sham group (*p <* 0.01, *n =* 5). A similar trend was noted in the hippocampal CA1 region, with percentages being approximately 8% for the atVNS group and around 13% for the Sham group (*p <* 0.05, *n =* 5). Such observations strongly advocate that atVNS effectively curtails the proliferation of astrocytes.

Further exploration using Western blot analysis provided insight into atVNS's capacity to regulate inflammation‐related signaling pathways (Figure 5E–I). Notably, atVNS led to a significant downregulation of TNF‐α expression levels in both the peri‐infarct cortex and hippocampal regions (*p <* 0.05 in both areas, *n =* 5). Concurrently, atVNS distinctly inhibited the phosphorylation levels of ERK and p38 MAPK. Specifically, in the peri‐infarct cortex area, the atVNS group demonstrated significantly lower p‐ERK/β‐actin and p‐p38 MAPK/β‐actin ratios compared to the Sham group (*p <* 0.01 and *p <* 0.001 respectively, *n =* 5). Similar patterns were discernible in the hippocampal area, where both p‐ERK and p‐p38 MAPK expression levels underwent significant decreases (both *p <* 0.001, *n =* 5). These findings propose that atVNS could potentially mitigate inflammation through the inhibition of ERK and p38 MAPK signaling pathways.

The validity of these Western blot results was further strengthened by corroborating immunofluorescent staining outcomes (Figure 5J–M). In both the peri‐infarct cortex and hippocampal CA1 regions, the p‐ERK fluorescent intensity was significantly subdued in the atVNS group when compared to the Sham group (Figure 5L, *p <* 0.05 in both areas, *n =* 5). Similarly, post‐atVNS treatment led to a significant decrease in the fluorescence intensity of p‐p38 MAPK (Figure 5M, peri‐infarct cortex: *p <* 0.01; hippocampus: *p <* 0.05, *n =* 5). Importantly, co‐localization analysis of p‐p38 MAPK and Iba‐1 indicates that this inhibitory effect predominantly transpires within activated microglia (Figure 5K).

### 3.6 atVNS Facilitated Post‐Stroke Upregulation of α7 Nicotine Acetylcholine Receptor (α7nAChR)

Previous research has identified the α7 nicotine acetylcholine receptor (α7nAChR) as a potentially critical target for the neuroprotective effects instigated by vagus nerve stimulation [28]. To delve deeper into the influence of atVNS on the expression of α7nAChR following cerebral ischemia, we undertook an investigation analyzing the expression of α7nAChR at distinct time intervals (D0, D7, and D28).

**FIGURE 6:**
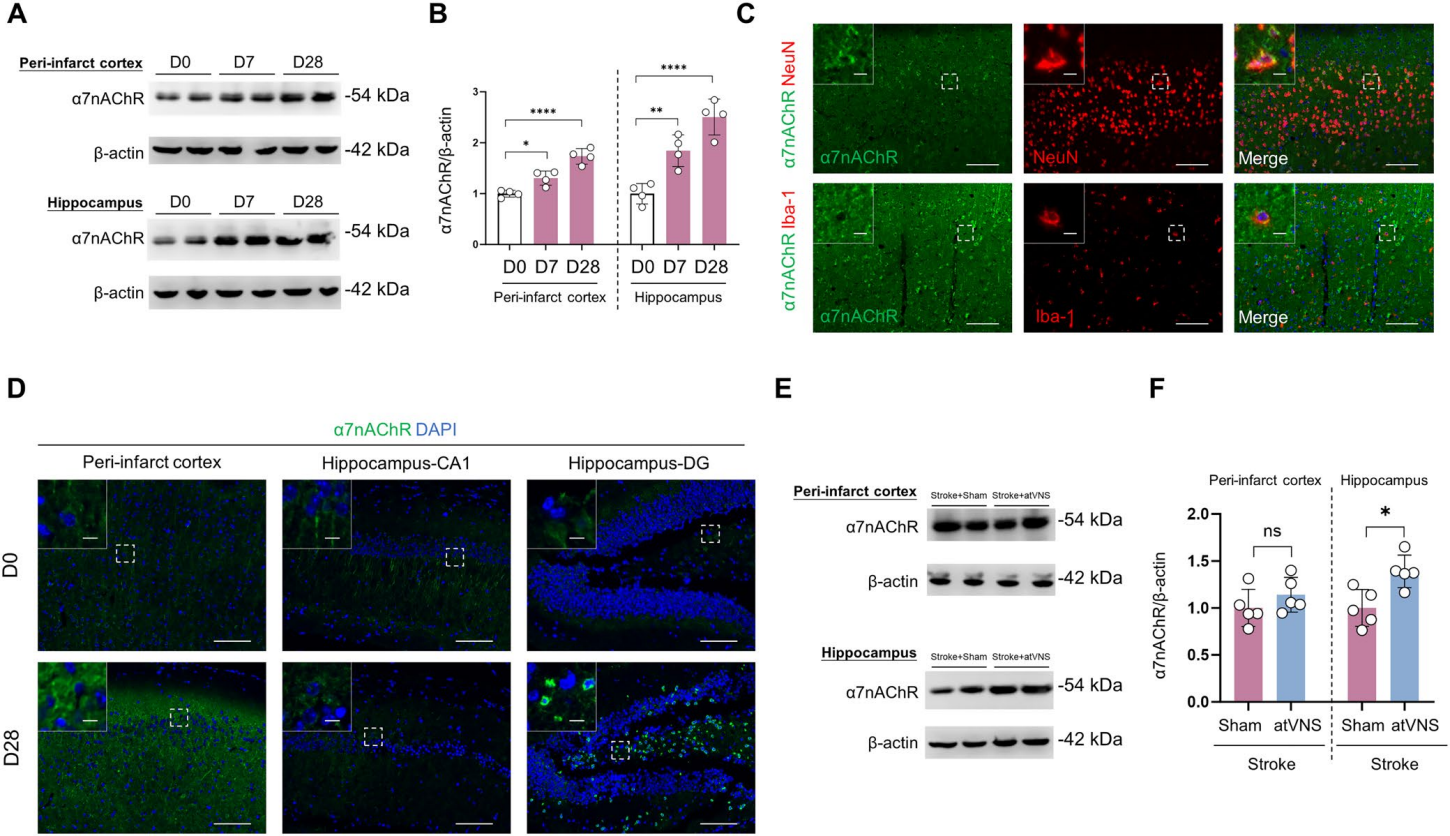
Expression of α7 nicotinic acetylcholine receptor (α7nAChR) after stroke and effects of auricular vagus nerve stimulation (atVNS). (A) Representative Western blots of α7nAChR in the peri‐infarct cortex and hippocampus at D0, D7, and D28 post‐stroke. (B) Quantification of α7nAChR/β‐Actin ratio from Western blots (*n =* 5 per group, *p < 0.05, **p < 0.01, ****p < 0.0001). (C) Representative immunofluorescence images showing co‐localization of α7nAChR (green) with NeuN (red, top) and Iba‐1 (red, bottom). Scale bars: 100 μm For main images, 10 μm for insets. (D) Representative immunofluorescence images of α7nAChR (green) in the peri‐infarct cortex, hippocampus‐CA1, and hippocampus‐DG regions at D0 and D28. DAPI (blue) labels nuclei. Scale bars: 100 μm For main images, 10 μm for insets. (E) Representative Western blots of α7nAChR in the peri‐infarct cortex and hippocampus for Stroke+Sham and Stroke+atVNS groups at D28. (F) Quantification of α7nAChR/β‐Actin ratio from Western blots in (E) (*n =* 5 per group, *p < 0.05, ns: Not significant). Data are presented as mean ± SD. Statistical analysis was performed using one‐way ANOVA with Tukey's *post hoc* test for (B) and unpaired t test for (F).

Our Western blot analysis disclosed a progressive escalation in the expression of α7nAChR over time within both the peri‐infarct cortex and the hippocampal regions (Figure 6A,B). Detailing this further, within the peri‐infarct cortex region, there was a marked increase in the α7nAChR/β‐actin ratio on D7 compared to the initial day (D0) (*p <* 0.0001, *n =* 5); this ratio rose even more significantly by D28 (*p <* 0.0001 vs. D0, *p <* 0.01 vs. D7, *n =* 5). This incremental trend was also observable in the hippocampal region with α7nAChR expression being notably higher on D28 than either D0 or D7 (*p <* 0.01, *n =* 5). Such findings imply that post‐cerebral ischemia, a dynamic amplification of α7nAChR expression can be observed.

Subsequent immunofluorescence staining corroborated the results gleaned from the Western blot analysis (Figure 6C,D). On comparison with D0, it was noted that the fluorescence intensity of α7nAChR had augmented appreciably within the peri‐infarct cortex, hippocampal CA1, and DG regions by D28. Interestingly, a colocalization study of α7nAChR and NeuN demonstrated that α7nAChR is mainly manifested within neurons (Figure 6C, top panels). Simultaneously, our studies showed no conspicuous colocalization between α7nAChR and Iba‐1 (a microglial cell marker) (Figure 6C, bottom panels), suggesting that the changes in the expression of α7nAChR primarily occur within neurons rather than microglial cells.

To assess the effect of atVNS on the protein expression of α7nAChR, we compared α7nAChR expression levels in both Stroke+Sham and Stroke+atVNS groups at D28 (Figure 6E,F). The Western blot outcomes revealed that, within the hippocampal region, the α7nAChR expression was significantly higher in the atVNS group compared to the Sham group (*p <* 0.05, *n =* 5). However, no statistical difference was detected in the α7nAChR expression levels between the two groups within the peri‐infarct cortex region (*p >* 0.05, *n =* 5). This indicates that atVNS could potentially exert a specific upregulating influence on α7nAChR expression within the hippocampal region.

### 3.7 Knockout of α7nAChR Diminishes the Capacity of atVNS to Inhibit Ferroptosis During the Post‐Stroke Recovery Period

**FIGURE 7:**
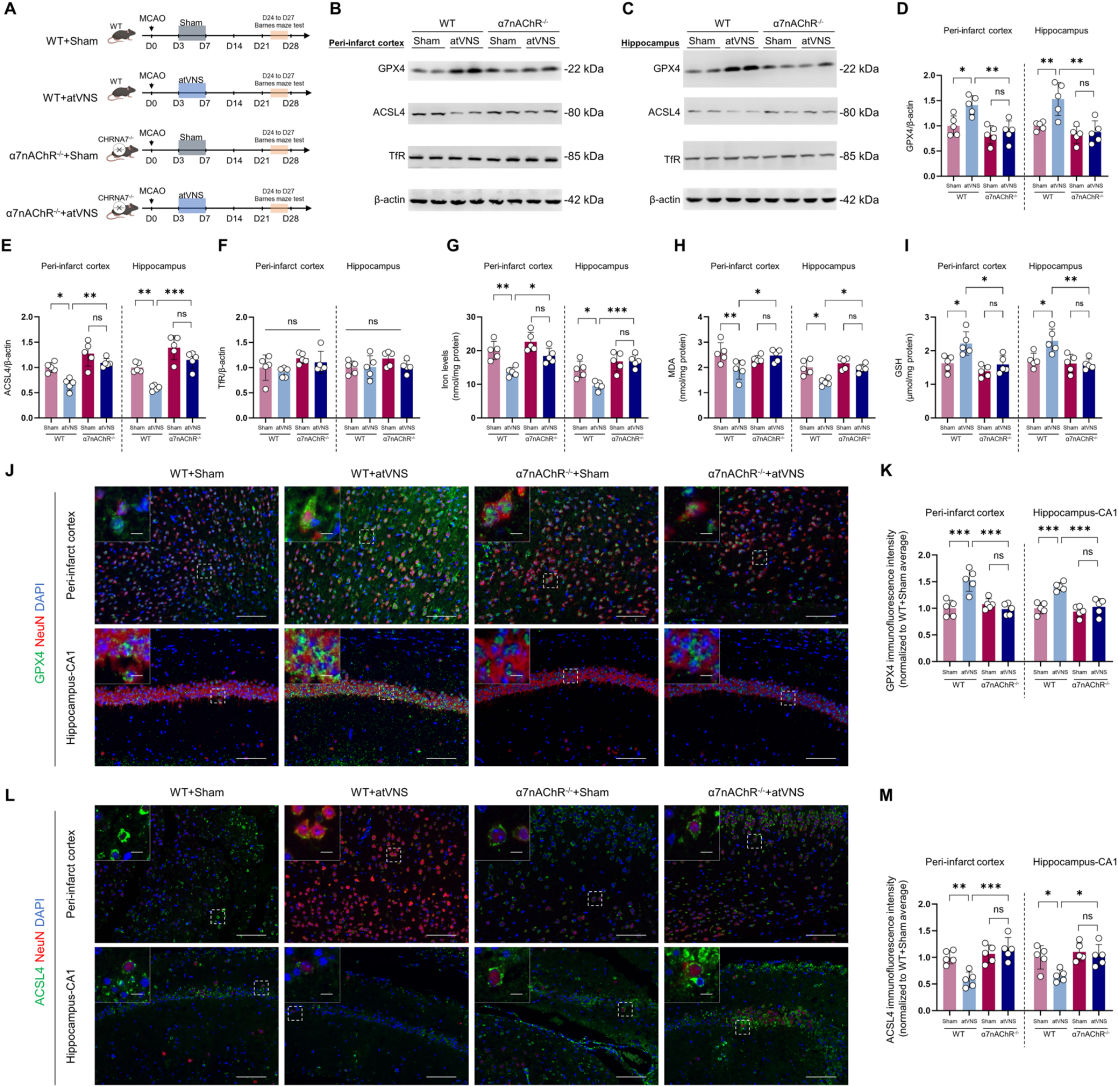
α7nAChR knockout diminishes the anti‐ferroptotic effects of atVNS during the Recovery Period After Stroke. (A) Experimental design for wild‐type (WT) and α7nAChR knockout (α7nAChR^−/−^) mice with MCAO and atVNS treatment. (B, C) Representative Western blots of GPX4, ACSL4, and TfR in the peri‐infarct cortex (B) and hippocampus (C) for all groups at D28. (D–I) Quantification of Western blot results for GPX4 (D), ACSL4 (E), TfR (F), and iron levels (G) in the peri‐infarct cortex and hippocampus, plus MDA (H) and GSH (I) levels (*n =* 5 per group, *p < 0.05, **p < 0.01, ***p < 0.001, ns: Not significant). (J) Representative immunofluorescence images of GPX4 (green) and NeuN (red) in the peri‐infarct cortex and hippocampus‐CA1. DAPI (blue) labels nuclei. Scale bars: 100 μm for main images, 10 μm for insets. (K) Quantification of GPX4 immunofluorescence intensity (*n =* 5 per group, ***p < 0.001, ns: Not significant). (L) Representative immunofluorescence images of ACSL4 (green) and NeuN (red) in the peri‐infarct cortex and hippocampus‐CA1. Scale bars: 100 μm for main images, 10 μm for insets. (M) Quantification of ACSL4 immunofluorescence intensity (*n =* 5 per group, *p < 0.05, **p < 0.01, ***p < 0.001, ns: Not significant). Data are presented as mean ± SD. Statistical analysis was performed using one‐way ANOVA with Tukey's post hoc test.

To elucidate the function of α7nAChR in the atVNS‐mediated suppression of ferroptosis after brain ischemia, we undertook a comparative research study involving wild‐type (WT) and α7nAChR knockout (α7nAChR^−/−^) mice. As Figure 7A depicts, our experimental design comprised four groups: WT + Sham, WT + atVNS, α7nAChR^−/−^ + Sham, and α7nAChR^−/−^ + atVNS. Each group underwent MCAO surgery on D0, with the atVNS group receiving treatments from D3 to D7. All indicators were measured on the 28th day after MCAO surgery.

Significant disparities were discerned by Western blot analysis in terms of how atVNS modifies the expression of proteins associated with ferroptosis in WT and α7nAChR^−/−^ mice (Figure 7B–D). Within the peri‐infarct cortex region, atVNS pronouncedly elevated the expression of GPX4 (*p <* 0.05, *n =* 5) and decreased ACSL4 (*p <* 0.01, *n =* 5) in WT mice on D28 after MCAO surgery. Interestingly, such modulation of GPX4 and ACSL4 by atVNS was absent in α7nAChR^−/−^ mice. Comparable trends were observed within the hippocampal zone. In WT mice, atVNS significantly intensified GPX4 (*p <* 0.01, *n =* 5) and mitigated ACSL4 expression (*p <* 0.001, *n =* 5), yet these effects were not significant in the α7nAChR^−/−^ mice. Of note, TfR expression did not present any significant variances across all groups (*p >* 0.05, *n =* 5).

Supporting this, iron level evaluations revealed that in WT mice, atVNS dramatically reduced the concentration of iron in both the peri‐infarct cortex and hippocampus areas (Figure 7E, both *p <* 0.01, *n =* 5). Conversely, the effect of atVNS on iron levels was statistically insignificant in α7nAChR^−/−^ mice (*p >* 0.05, *n =* 5), indicating that α7nAChR might play an essential role in the atVNS‐induced regulation of iron metabolism.

Further analyses of oxidative stress biomarkers revealed significant changes (Figure 7H,I). In the peri‐infarct cortex, atVNS treatment significantly decreased MDA levels (*p <* 0.01, *n =* 5) and increased GSH levels (*p <* 0.05, *n =* 5) in WT mice. Similar results were observed in the hippocampus, where atVNS significantly reduced MDA levels (*p <* 0.05, *n =* 5) and elevated GSH levels (*p <* 0.01, *n =* 5) in WT mice. Notably, these antioxidative effects of atVNS were absent in α7nAChR^−/−^ mice (*p >* 0.05, *n =* 5), further emphasizing the crucial role of α7nAChR in mediating the antioxidant effects of atVNS after brain ischemia.

Immunofluorescence staining (Figure 7J–M) demonstrated that in WT mice' peri‐infarct cortex and CA1 region of the hippocampus, the fluorescence intensity of GPX4 was significantly higher in the atVNS‐treated group than the Sham group (both *p <* 0.001, *n =* 5). In contrast, atVNS treatment showed a significant decrease in ACSL4 fluorescence intensity (peri‐infarct cortex: *p <* 0.01; hippocampal CA1 region: *p <* 0.05, *n =* 5). However, these modulatory impacts of atVNS on GPX4 and ACSL4 expression were not significant in α7nAChR^−/−^ mice (*p >* 0.05, *n =* 5).

Together, these findings advocate for the critical role of α7nAChR in the atVNS‐driven inhibition of ferroptosis during the chronic recovery phase following stroke. The absence of α7nAChR significantly blunts or nullifies the regulatory effects of atVNS on GPX4 and ACSL4 expression, as well as its impact on iron levels, MDA, and GSH, highlighting the α7nAChR‐dependent antioxidant and anti‐ferroptotic mechanisms of atVNS therapy.

### 3.8 α7nAChR Knockout Diminishes atVNS‐Aided Neurological and Cognitive Function Recovery Following Stroke

**FIGURE 8:**
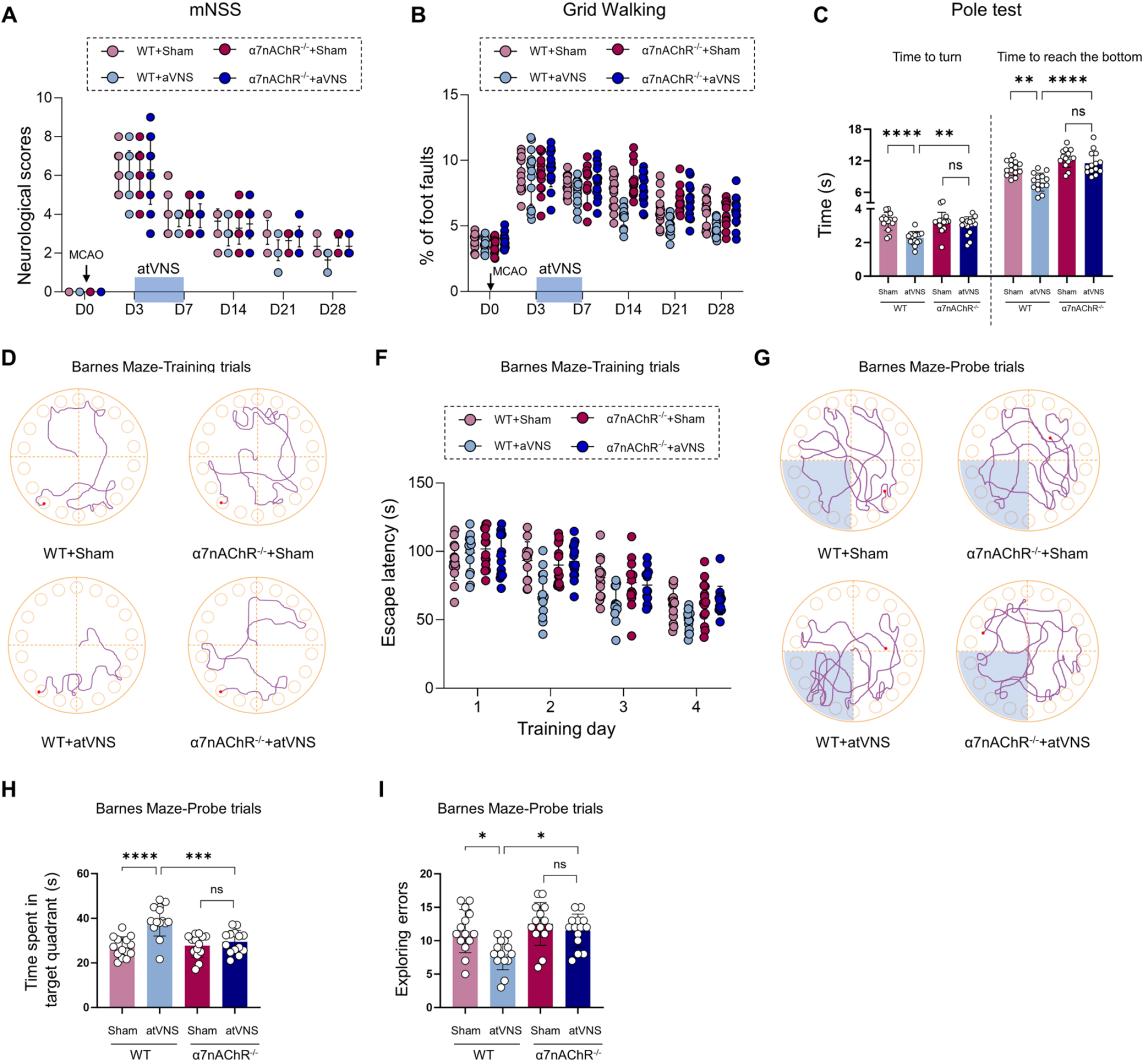
Effects of α7nAChR knockout on atVNS‐induced recovery of cognitive and neurological functions post‐stroke. (A) Variation in Modified Neurological Severity Score (mNSS) across time for all groups (*n =* 14 each). (B) Grid walking test results indicating percentage of foot faults over time (*n =* 14 per group). (C) Results of pole test showing time taken to turn and reach the bottom (*n =* 14 per group, **p < 0.01, ****p < 0.0001, ns: Not significant). (D) Illustrative trajectories of mice during Barnes Maze training trials. (F) Latency to escape during Barnes Maze training trials across 4 days (*n =* 14 per group). (G) Representational tracks of mice during Barnes Maze probe trials. (H) Duration in target quadrant during Barnes Maze probe trials (*n =* 14 per group, ***p < 0.001, ****p < 0.0001, ns: Not significant). (I) Count of exploratory errors during Barnes Maze probe trials (*n =* 14 per group, *p < 0.05, ns: Not significant). Data is shown as mean ± SD. Statistical analysis was utilized using two‐way ANOVA with Tukey's *post hoc* test for A, B, F and one‐way ANOVA with Tukey's *post hoc* test for C, H, I.

The neurological function assessment demonstrates that atVNS plays a significant role in enhancing the recovery of neurological functions in WT mice. However, this beneficial effect appears to diminish markedly in α7nAChR^−/−^ mice. The mNSS scores further corroborate these findings, revealing a more rapid post‐treatment recovery rate in the WT + atVNS group compared to the WT + Sham cohort (Figure 8A, *p <* 0.05, *n =* 14). Interestingly, there is no discernible difference between the two α7nAChR^−/−^ groups. Concordantly, Grid walk tests also exhibited a similar trend, with the foot fault rate decreasing more rapidly in the WT + atVNS group, whereas the effect of atVNS in α7nAChR^−/−^ mice is not prominent (Figure 8B, *p >* 0.05, *n =* 14).

The Pole test presents additional evidence underlining the integral role of α7nAChR in the atVNS‐facilitated enhancement of motor function recovery (Figure 8C). In the case of WT mice, atVNS significantly expedited both the turn‐around time (*p <* 0.01, *n =* 14) and the time taken to reach the base (*p <* 0.0001, *n =* 14). Yet, for α7nAChR^−/−^ mice, atVNS did not exhibit any meaningful influence on these parameters (*p >* 0.05, *n =* 14).

The Barnes Maze test was deployed to assess spatial learning and memory capacity. During the training phase, the WT + atVNS group demonstrated the most pronounced reduction in escape latency, thereby indicating superior learning efficiency (Figure 8D,F). The performance of the α7nAChR^−/−^ + atVNS group, however, paralleled that of the α7nAChR^−/−^ + Sham group, with both falling notably short of the WT + atVNS group's achievements (*p >* 0.05, *n =* 14). A subsequent probe trial confirmed these findings, with the WT + atVNS group registering both the longest stay in the target quadrant (Figure 8H, *n =* 14, *p <* 0.0001) and the fewest exploration errors (Figure 8I, *p <* 0.05). By contrast, these atVNS effects were not statistically impactful in α7nAChR^−/−^ mice (Figure 8G–I, *p >* 0.05, *n =* 14).

### 3.9 α7nAChR Deletion Attenuates atVNS‐Induced Augmentation of Neurogenesis and Angiogenesis in Post‐Stroke Recovery

**FIGURE 9:**
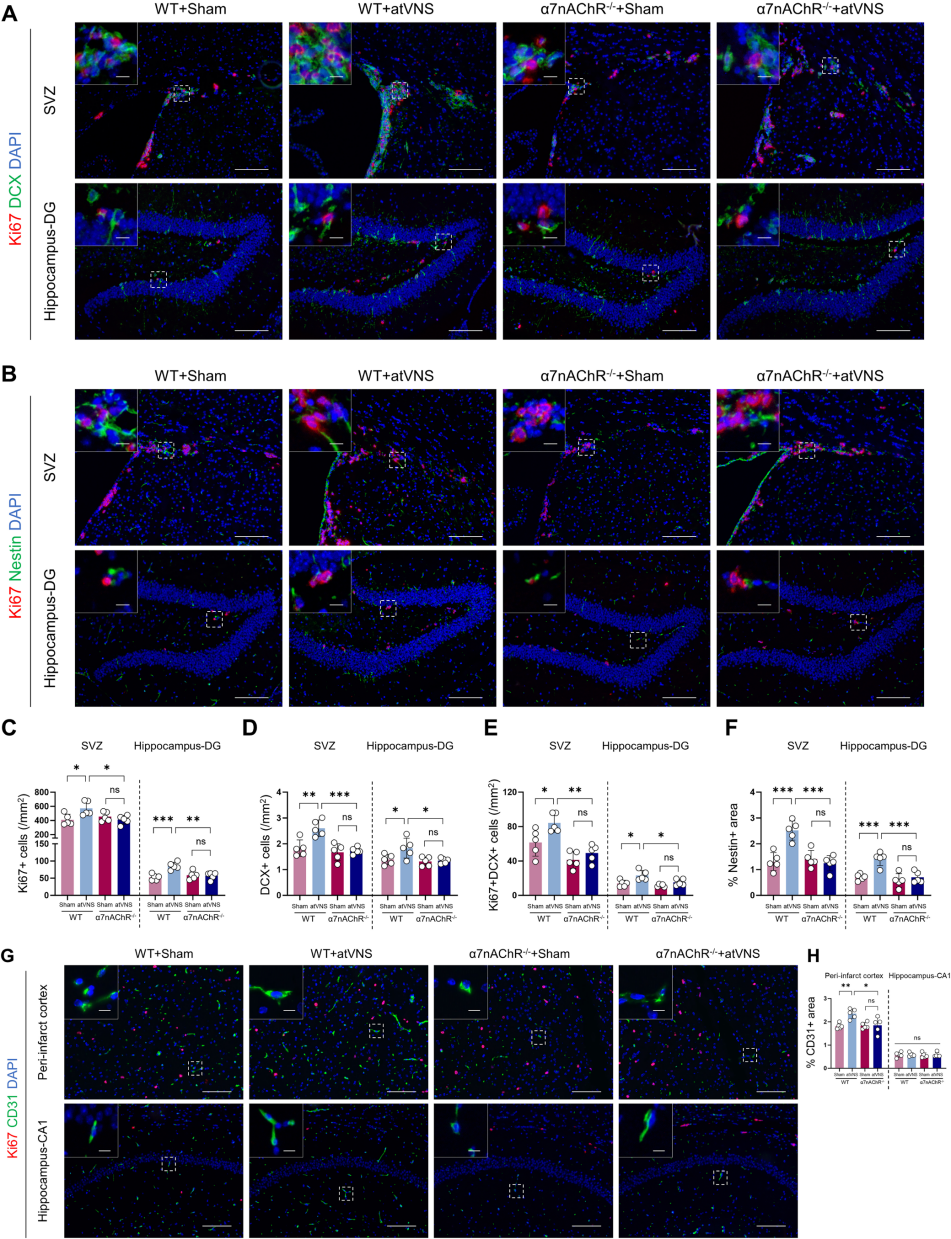
Elimination of α7nAChR curbs the enhancement of neurogenesis and angiogenesis instigated by atVNS. (A) Representative immunofluorescence images of Ki67 (red), DCX (green), and DAPI (blue) in the subventricular zone (SVZ) and hippocampus dentate gyrus (DG) regions. Scale bars: 100 μm for main images, 10 μm for insets. (B) Representative immunofluorescence images of Ki67 (red), Nestin (green), and DAPI (blue) in the SVZ and hippocampus DG regions. Scale bars: 100 μm for main images, 10 μm for insets. (C) Quantification of Ki67+ cells in the SVZ and hippocampus DG (*n =* 5 per group, *p < 0.05, **p < 0.01, ***p < 0.001, ns: Not significant). (D) Quantification of DCX+ cells in the SVZ and hippocampus DG (*n =* 5 per group, *p < 0.05, **p < 0.01, ***p < 0.001, ns: Not significant). (E) Quantification of Ki67+/DCX+ double‐positive cells in the SVZ and hippocampus DG (*n =* 5 per group, *p < 0.05, **p < 0.01, ns: Not significant). (F) Quantification of Nestin+ area in the SVZ and hippocampus DG (*n =* 5 per group, ***p < 0.001, ns: Not significant). (G) Representative immunofluorescence images of Ki67 (red), CD31 (green), and DAPI (blue) in the peri‐infarct cortex and hippocampus CA1 regions. Scale bars: 100 μm for main images, 10 μm for insets. (H) Quantification of CD31+ area in the peri‐infarct cortex and hippocampus CA1 regions (*n =* 5 per group, *p < 0.05, **p < 0.01, ns: Not significant). Data are presented as mean ± SD. Statistical analysis was performed using one‐way ANOVA with Tukey's *post hoc* test.

Immunofluorescence staining results provided significant evidence of atVNS promoting neurogenesis in WT mice, although this effect was considerably attenuated in α7nAChR^−/−^ mice (Figure 9A,B). In the context of WT mice, there was a notable increase in Ki67+ cell count post‐atVNS treatment (Figure 9C). This proliferative effect manifested prominently in both subventricular zone (SVZ; *p <* 0.05, *n =* 5) and dentate gyrus (DG; *p <* 0.001, *n =* 5) regions. Conversely, the impact of atVNS on Ki67+ cell count in α7nAChR^−/−^ mice did not reach statistical significance (*p >* 0.05, *n =* 5).

Further scrutiny into neuronal precursor cells, represented by DCX+ cell quantity (Figure 9D), revealed that atVNS significantly elevated these counts in both SVZ (*p <* 0.01, *n =* 5) and DG (*p <* 0.001, *n =* 5) within WT mice. However, such an effect was markedly lessened in α7nAChR^−/−^ mice (*p >* 0.05, *n =* 5). Examining the Ki67+/DCX+ double‐positive cells, indicative of proliferating neuronal precursor cells (Figure 9E), it was observed that atVNS led to a substantial increase in their numbers within WT mice's SVZ (*p <* 0.05, *n =* 5) and DG (*p <* 0.01, *n =* 5). Yet, such an effect was unremarkable in α7nAChR^−/−^ mice (*p >* 0.05, *n =* 5). Nestin+ area, reflecting the abundance of neural stem cells (Figure 9F), was drastically heightened in both SVZ and DG following atVNS treatment in WT mice (*p <* 0.001, *n =* 5). Nevertheless, a comparable surge was absent in α7nAChR^−/−^ mice (*p >* 0.05, *n =* 5).

Moreover, angiogenesis, as assessed through the CD31+ area surrounding the infarcted cortex (*p <* 0.01) and hippocampal CA1 region (*p <* 0.05), demonstrated an increased vascular density subsequent to atVNS application in WT mice (Figure 9G,H). Regrettably, this pro‐angiogenic response to atVNS was notably curtailed in α7nAChR^−/−^ mice (*p >* 0.05, *n =* 5).

### 3.10 Knockout of α7nAChR Diminishes Neuroinflammatory Protection Induced by atVNS in Post‐Stroke Recovery

**FIGURE 10:**
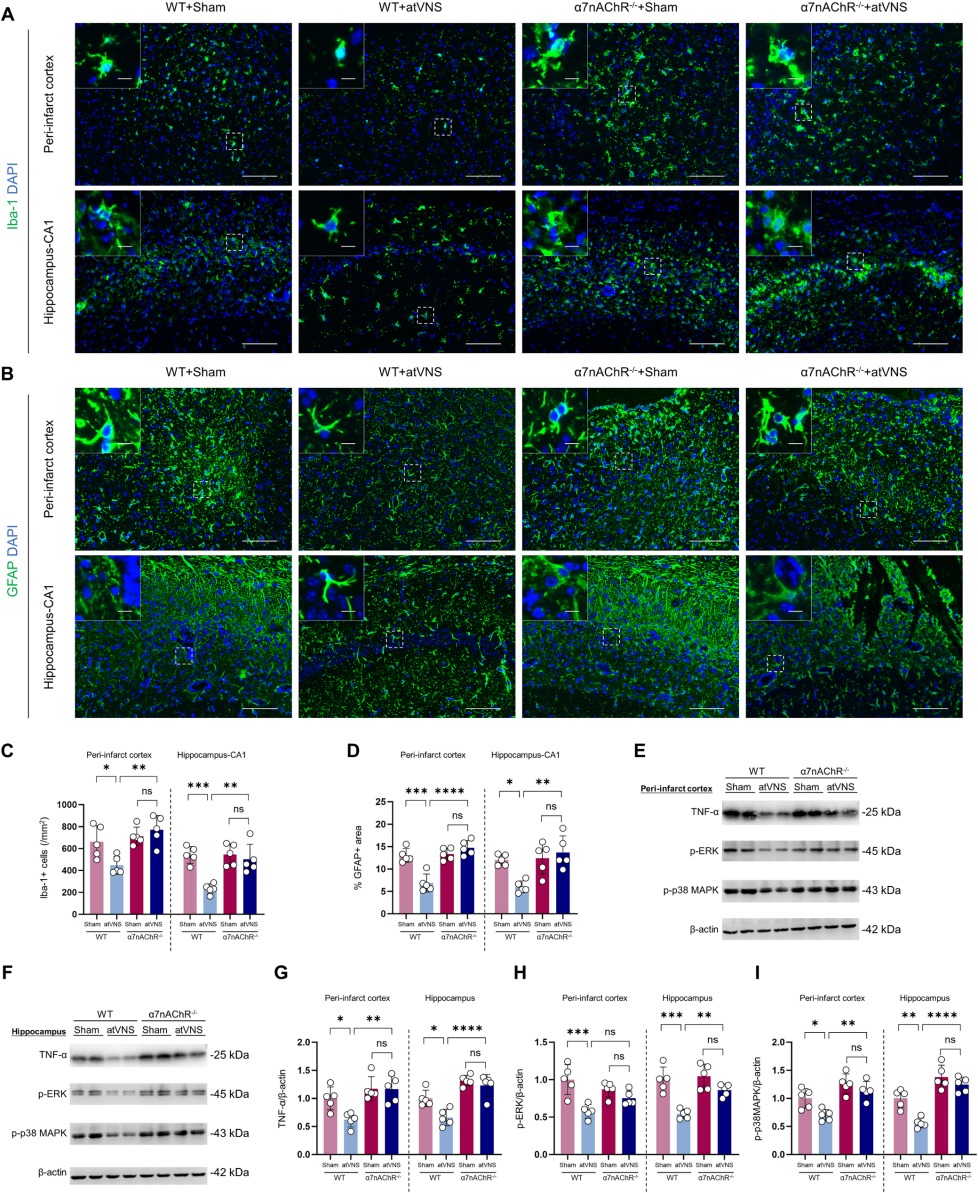
α7nAChR Knockout diminishes atVNS‐induced neuroinflammatory protection after stroke. (A) Representative immunofluorescence images of Iba1 (green) and DAPI (blue) in the peri‐infarct cortex and hippocampus CA1 regions. Scale bars: 100 μm For main images, 10 μm for insets. (B) Representative immunofluorescence images of GFAP (green) and DAPI (blue) in the peri‐infarct cortex and hippocampus CA1 regions. Scale bars: 100 μm For main images, 10 μm for insets. (C) Quantification of Iba1+ cells in the peri‐infarct cortex and hippocampus CA1 (*n =* 5 per group, *p < 0.05, **p < 0.01, ***p < 0.001, ns: Not significant). (D) Quantification of GFAP+ area in the peri‐infarct cortex and hippocampus CA1 (*n =* 5 per group, *p < 0.05, **p < 0.01, ***p < 0.001, ****p < 0.0001, ns: Not significant). (E, F) Representative Western blot images of TNF‐α, p‐ERK, p‐p38 MAPK, and β‐Actin in the peri‐infarct cortex (E) and hippocampus (F). (G–I) Quantification of Western blot results for TNF‐α (G), p‐ERK (H), and p‐p38 MAPK (I) in the peri‐infarct cortex and hippocampus (*n =* 5 per group, *p < 0.05, **p < 0.01, ***p < 0.001, ****p < 0.0001, ns: Not significant). Data are presented as mean ± SD. Statistical analysis was performed using one‐way ANOVA with Tukey's *post hoc* test.

The findings derived from immunofluorescence staining results (Figure 10A,B) revealed that atVNS significantly attenuated the activation of microglial cells and hindered the proliferation of astrocytes in WT mice. Nevertheless, this inhibitory effect was notably diminished in α7nAChR^−/−^ mice (*p >* 0.05, *n =* 5).

The level of microglial activation was gauged by quantifying Iba1‐positive cells (Figure 10C). Among WT mice, atVNS meaningfully reduced the number of Iba1‐positive cells located both within the periphery of the infarct cortex (*p <* 0.05) and the hippocampal CA1 region (*p <* 0.001). Conversely, the impact of atVNS on the number of Iba1‐positive cells was not statistically significant in the α7nAChR^−/−^ mice (*p >* 0.05, *n =* 5).

Astrocyte proliferation was assessed by observing the area of GFAP‐positive regions (Figure 10D). In WT mice, atVNS led to a significant decrease in GFAP‐positive areas in the infarct periphery cortex (*p <* 0.001) and the hippocampal CA1 region (*p <* 0.01). Contrarily, the α7nAChR^−/−^ mice exhibited a stark reduction in this atVNS‐induced effect (*p >* 0.05, *n =* 5).

Western blot analysis (Figure 10E–I) further corroborated the modulatory influence of atVNS on the expression of inflammation‐associated proteins. Specifically, in the infarct periphery cortex, atVNS distinctly curtailed the expression of TNF‐α in WT mice (*p <* 0.05, *n =* 5), an effect absent in the α7nAChR^−/−^ mice (*p >* 0.05, *n =* 5). Similarly, in the hippocampus, atVNS markedly downregulated TNF‐α expression in WT mice (*p <* 0.01, *n =* 5), but this effect was nonsignificant in α7nAChR^−/−^ mice (*p >* 0.05, *n =* 5). With respect to p‐ERK expression, its levels were considerably lowered in the infarct periphery cortex and hippocampal region of WT mice following atVNS (both *p <* 0.001, *n =* 5); such an effect, however, was not observed in α7nAChR^−/−^ mice. Parallel trends were observed for p‐p38 MAPK expression, with atVNS significantly decreasing its expression in the infarct periphery cortex and hippocampal regions of WT mice (*p <* 0.01 and *p <* 0.05, *n =* 5), but exerting no significant impact on α7nAChR^−/−^ mice (*p >* 0.05, *n =* 5).

## 4 Discussion

Our research offers a comprehensive examination of the spatiotemporal dynamics of ferroptosis during the post‐ischemic stroke recovery phase, with an added focus on understanding the regulatory role of auricular vagus nerve stimulation (atVNS) within this context. We reveal dynamic changes in proteins associated with ferroptosis from the acute to chronic stages of post‐stroke recovery. Specifically, GPX4 expression in the cortex steadily diminishes over time, while in the hippocampus, it decreases initially before gradually increasing again. Conversely, both ACSL4 and TfR proteins consistently increase in both regions. We further establish that atVNS can effectively mitigate the progression of ferroptosis, as indicated by GPX4 upregulation and ACSL4 downregulation. In addition, our study identifies supplementary benefits of atVNS, such as enhanced neurogenesis and angiogenesis, and suppressed activation of glial cells and inflammatory signaling pathways. By utilizing α7 nicotinic acetylcholine receptor (α7nAChR) gene knockout mice, we demonstrate that the absence of α7nAChR significantly impairs the therapeutic efficacy of atVNS, thus underlining its critical role in atVNS‐mediated neuroprotection.

While previous studies have highlighted ferroptosis's crucial involvement during the acute phase of ischemic stroke, asserting that ferroptosis prohibition can remarkably reduce infarct volume [8, 29, 30], our investigation extends this understanding into the broader continuum of post‐stroke recovery. We document perpetual alterations in the ferroptosis‐associated protein expressions during chronic phases, a phenomenon that has also been recently observed in myocardial infarction recovery, where persistent ferroptosis significantly influences cardiac remodeling and inflammatory responses [11]. GPX4 protein, integral to ferroptosis regulation, serves a protective function against ferroptosis by impeding lipid peroxidation, maintaining iron homeostasis, and participating in comprehensive antioxidant defenses [31]. Moreover, it has been observed that GPX4 levels generally decline in brain tissue amid acute ischemic stroke events [8, 29]. Of significant interest is the regional‐specific transformation in GPX4 expression. In our study, a time‐dependent pattern of initial decrease followed by a subsequent increase was discerned in the hippocampal expression of GPX4, hinting at a potential compensatory mechanism specific to this region.

Moreover, we unveil a consistent elevation in ACSL4 and TfR expressions, along with the concentration of iron ions during the post‐stroke recovery phase. The observations align with those reported by Guo et al. [12], who mentioned persistent above‐baseline expressions of TfR and elevated iron ion concentrations, even 28 days after the stroke. ACSL4, instrumental to ferroptosis progression, engenders the activation of long‐chain polyunsaturated fatty acids (PUFAs), specifically arachidonic acid (AA) and adenosine acid (AdA), thereby magnifying cell membrane sensitivity to ferroptosis [8, 32]. TfR, however, serves as the primary pathway for cellular iron uptake, mirroring the cell's need for iron [33]. During ferroptosis, TfR‐mediated iron uptake might exacerbate intracellular iron accumulation, facilitating ferroptosis [34]. Our electron microscopy findings, revealing neurons adjacent to the ischemic lesion exhibiting features consistent with ferroptosis even 28 days after MCAO, further solidify the notion that ferroptosis and its related signaling pathways persist in their activity throughout the stroke recovery phase.

While considerable research supports the protective role of VNS on cerebral ischemia [35, 36], its specific influence on ferroptosis has remained unexplored until our present investigation. We discovered that, during the subacute phase post‐stroke (3–7 days following MCAO), atVNS not only facilitates neural function recovery but also effectively modulates key proteins associated with ferroptosis throughout the stroke recovery period (28 days post‐MCAO surgery). This modulation is specifically characterized by the upregulation of GPX4 and downregulation of ACSL4 within ischemic penumbra tissues in mice post‐stroke. These findings suggest that atVNS treatment administered during the subacute phase of stroke could yield enduring neuroprotective effects.

Contrarily, we found that the impact of atVNS on TfR and iron ion concentration was relatively insignificant, implying that the neuroprotective effect of atVNS does not derive from an alteration in global iron metabolism. Instead, it may be achieved by adjusting intracellular molecular structures, thereby managing the sensitivity to iron toxicity. In the aftermath of a stroke, along with motor‐sensory function decline, cognitive impairment constitutes another significant issue [37, 38]. Interestingly, we observed that atVNS extends its influence beyond just the tissue surrounding the ischemic foci, eliciting comparable effects within the hippocampus. In behavioral terms, this is reflected as improved performance in the Barnes Maze task. Thus, our research suggests that the benefits of atVNS might be extensive, influencing not solely the directly affected regions but also potentially incorporating areas related to cognitive function and memory.

Numerous studies have posited that the activation of the cholinergic anti‐inflammatory pathway, facilitated predominantly by α7nAchR, is an integral mechanism through which VNS provides neuroprotective effects in the context of ischemic injury [39, 40]. Specifically, α7nAchR's activity and upregulation can be induced by endogenous acetylcholine stimulated by VNS [41]. However, literature documenting changes in α7nAchR during the chronic recovery phase following stroke remains absent. To address this gap, our study investigated the changes in α7nAchR levels within the ischemic cortex and hippocampus, revealing a significant elevation compared to pre‐ischemia baseline levels on both the 7th and 28th days post‐stroke. Given the established neuroprotective role of α7nAchR, these results suggest that its upregulation may be interpreted as a self‐protective response of the nervous system to ischemic injury.

Additionally, while some studies suggest that under inflammatory conditions, α7nAchR can express in microglia and exert anti‐inflammatory effects [17, 42], our findings contrastingly indicate that during the stroke recovery period, α7nAchR primarily manifests within neurons rather than microglial cells across both the cortex and hippocampus. This aligns with prior in situ hybridization and immunohistochemical investigations demonstrating that α7nAChR principally resides in neurons, particularly within cortical and hippocampal regions [43, 44].

Our research provides further evidence that atVNS can alleviate neuroinflammation during stroke recovery. Specifically, atVNS exerts a substantial inhibitory impact on neuroinflammation, as indicated by the deactivation of microglia, reduction in astrocyte proliferation, and the downregulation of inflammation‐related signaling pathways such as p38MAPK and ERK. However, the scenario significantly changes in α7nAChR knockout mice, wherein the ability of atVNS to inhibit microglial activation, astrocyte proliferation, and the expression of inflammation‐associated proteins is notably diminished. This observation underscores the pivotal role of α7nAChR in the counteractive response of atVNS against post‐stroke neuroinflammation. Due to our finding that α7nAChR exhibits primary expression in neurons rather than microglia, it may hint that atVNS modulates the activity of microglia and astrocytes indirectly by stimulating α7nAChR present in neurons.

Furthermore, we observed a marked reduction in the suppressive action of atVNS on ferroptosis in α7nAChR knockout mice. This suggests a close association between the mitigative influence of atVNS on post‐stroke ferroptosis and α7nAChR. α7nAChR might be directly implicated in the regulation of ferroptosis. According to extant research, the activation of α7nAChR can fortify the antioxidant defense system and initiate antioxidant signaling pathways such as HO‐1 [45]. Given that ferroptosis represents an oxidative stress‐driven form of cell death, the activation of α7nAChR could potentially inhibit ferroptosis through the enhancement of antioxidant capabilities. In addition, there is a sophisticated interplay between ferroptosis and inflammation where an inflammatory response can expedite ferroptosis [46, 47], while ferroptosis itself can incite an inflammatory response [48, 49]. Considering these observations, it seems likely that the anti‐inflammatory activity mediated by α7nAChR constitutes a crucial mechanism underpinning the inhibitory effect of atVNS on ferroptosis. As a result, in α7nAChR knockout mice, the diminished anti‐inflammatory capability of atVNS might indirectly contribute to a decrease in its effectiveness against ferroptosis.

Our analysis also highlights the essential function of atVNS in promoting neurogenesis and angiogenesis following ischemic stroke. It has been observed that atVNS not only increases the number and proliferative capacity of neural stem cells (Nestin+) and neural progenitor cells (DCX+), but also stimulates the proliferation of vascular endothelial cells. These outcomes align with previous studies that suggest the role of vagus nerve stimulation in enhancing neuroplasticity and fostering neurogenesis [50, 51]. In the scenario of α7nAChR gene‐knockout mice, the stimulatory effect of atVNS on neural stem cell proliferation, neural progenitor cell differentiation, and vascular endothelial cell proliferation was found to be significantly diminished. This affirmatively confirms the pivotal role of α7nAChR in atVNS‐mediated neurogenesis and angiogenesis processes. Based on our earlier research demonstrating that atVNS inhibits ferroptosis and neuroinflammation, we hypothesize that atVNS may indirectly foster neurogenesis and angiogenesis through α7nAChR‐mediated anti‐inflammatory and antioxidant pathways. Concurrently, this could enhance the local microenvironment by encouraging neurogenesis and angiogenesis, thereby initiating a positive feedback loop beneficial for neural repair.

Our study has several important limitations that warrant acknowledgment. Firstly, the exclusive use of male subjects prevents us from examining potential sexual dimorphism in α7nAChR responses to atVNS treatment. Given the well‐documented sex differences in stroke incidence, progression, and recovery, future studies should include both male and female subjects to provide a more comprehensive understanding of atVNS efficacy across biological sexes. Secondly, our study's temporal scope was limited to 28 days post‐stroke, which, while providing valuable insights into acute and early subacute recovery phases, does not capture potential long‐term effects of atVNS treatment. This absence of data beyond this period limits our ability to assess the sustained impact of the intervention on functional recovery and neuroplasticity. To strengthen the translational framework, future investigations should address these gaps by including both sexes, extending the follow‐up period beyond 28 days, and considering age as a variable by incorporating both young and aged animal models to better reflect the clinical stroke population.

## 5 Conclusions

**FIGURE 11:**
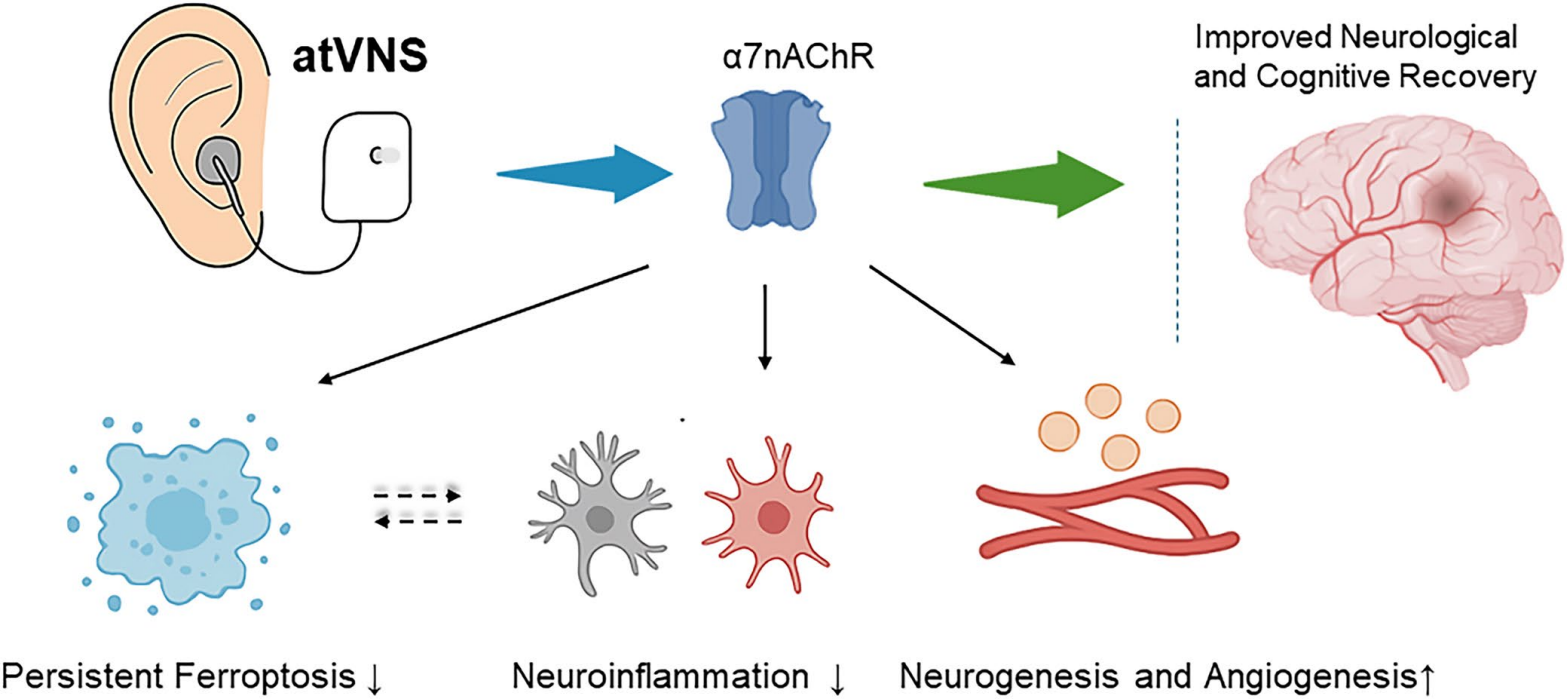
Proposed mechanisms mediating the therapeutic effects of atVNS during the stroke recovery. Activation of the α7nAChR by atVNS is hypothesized to trigger several beneficial pathways: (1) inhibition of ferroptosis, evidenced by increased GPX4 expression and decreased ACSL4 levels and lipid peroxidation; (2) suppression of neuroinflammation, mediated via reduced glial (microglia and astrocyte) activation and downregulation of pro‐inflammatory signaling; and (3) stimulation of neurogenesis and angiogenesis. Crucially, ferroptosis and neuroinflammation are tightly interlinked; ferroptotic cell death can release damage‐associated molecular patterns (DAMPs) and oxidized lipids that propagate inflammatory responses, while inflammatory mediators released by activated glia can, in turn, sensitize neurons to ferroptosis by increasing oxidative stress or altering iron metabolism. Therefore, the simultaneous targeting of both processes by atVNS via α7nAChR may disrupt this detrimental feedback loop. These combined therapeutic effects ultimately contribute to improved neurological and cognitive recovery.

Our investigation reveals significant insights into post‐ischemic stroke recovery and the therapeutic potential of atVNS. We demonstrate that ferroptosis persists beyond the acute stroke phase into the chronic recovery period, similar to observations in other ischemic conditions, suggesting its broader significance in tissue repair processes. Importantly, we show that atVNS effectively modulates ferroptosis‐associated proteins while promoting neurogenesis, angiogenesis, and reducing neuroinflammation, with these effects primarily mediated through α7nAChR (Figure 11). These findings challenge traditional views of stroke pathophysiology and establish atVNS as a promising noninvasive adjunctive treatment. The identification of α7nAChR as a crucial mediator not only explains the comprehensive therapeutic effects of atVNS but also provides a foundation for developing targeted interventions. Our research thus opens new avenues for improving long‐term outcomes in stroke patients, with potential implications for treating other ischemic conditions.

## Author Contributions

**Hongyan Gong:** investigation, formal analysis, writing‐original draft preparation. **Fang Zheng:** validation, methodology. **Bochao Niu:** investigation. **Bin Wang:** investigation, methodology. **Lin Xu:** investigation. **Yunchao Yang:** investigation, methodology. **Jiahan Wang:** writing – review and editing, conceptualization. **Xiaopeng Tang:** funding acquisition, methodology. **Yanlin Bi:** writing – review and editing, supervision, conceptualization.

## Conflicts of Interest

The authors declare no conflicts of interest.

## Data Availability

All datasets produced or analyzed during this study can be obtained from the corresponding author upon reasonable request.
